# Increased long noncoding RNA LINK-A contributes to rheumatoid synovial inflammation and aggression

**DOI:** 10.1172/jci.insight.146757

**Published:** 2021-12-08

**Authors:** Jingnan Wang, Chuyu Shen, Ruiru Li, Cuicui Wang, Youjun Xiao, Yu Kuang, Minxi Lao, Siqi Xu, Maohua Shi, Xiaoyan Cai, Liuqin Liang, Hanshi Xu

**Affiliations:** 1 Department of Rheumatology and Immunology, The First Affiliated Hospital, Sun Yat-sen University, Guangzhou, China.; 2 Department of Rheumatology, Guangzhou First People’s Hospital, School of Medicine, South China University of Technology, Guangzhou, China.

**Keywords:** Immunology, Noncoding RNAs, Rheumatology

## Abstract

Fibroblast-like synoviocytes (FLSs) play a key role in controlling synovial inflammation and joint destruction in rheumatoid arthritis (RA). The contribution of long noncoding RNAs (lncRNAs) to RA is largely unknown. Here, we show that the lncRNA LINK-A, located mainly in cytoplasm, has higher-than-normal expression in synovial tissues and FLSs from patients with RA. Synovial LINK-A expression was positively correlated with the severity of synovitis in patients with RA. LINK-A knockdown decreased migration, invasion, and expression and secretion of matrix metalloproteinases and proinflammatory cytokines in RA FLSs. Mechanistically, LINK-A controlled RA FLS inflammation and invasion through regulation of tyrosine protein kinase 6–mediated and leucine-rich repeat kinase 2–mediated HIF-1α. On the other hand, we also demonstrate that LINK-A could bind with microRNA 1262 as a sponge to control RA FLS aggression but not inflammation. Our findings suggest that increased level of LINK-A may contribute to FLS-mediated rheumatoid synovial inflammation and aggression. LINK-A might be a potential therapeutic target for RA.

## Introduction

Rheumatoid arthritis (RA) is a common autoimmune disease characterized by chronic synovial inflammation and progressive articular damage (1). Fibroblast-like synoviocytes (FLSs), the active resident cell component in synovial intimal lining, play a critical role in aggressive action of synovial pannus tissue (2). Stable activated RA FLSs exhibit not only increased secretion of inflammatory mediators but also abnormal, aggressive behavior. It has been found that RA FLSs are epigenetically imprinted with an aggressive phenotype. Targeting activated FLS aggression is an attractive therapeutic approach for controlling joint damage of RA (3, 4). Accumulating evidence suggests autonomous factors including epigenetic changes may have important roles in development of the aggressive phenotype of RA FLSs (5).

The majority of the mammalian genome is transcribed to noncoding RNAs (ncRNAs), and ncRNAs are important factors in modulating the physiological functions of mammalian cells (6, 7). It is well-known that ncRNAs are classified into 2 groups based on their length. One group is long noncoding RNAs (lncRNAs), which are defined as ncRNAs longer than 200 nucleotides (nt) without protein coding ability, and the other group is microRNAs (miRNAs), whose length is between 20 and 24 nt. Both lncRNAs and miRNAs have been demonstrated to be crucial regulators of gene expression at the epigenetic, transcriptional, and posttranscriptional levels and can actively participate in various physiological and pathological processes (8, 9). For instance, lncRNA is required for mouse acute leukemia maintenance by promoting leukemia stem cell signatures (10). LncRNA NEAT1 was proved to promote glioma cells’ growth and invasiveness by promoting SOX2 expression through suppressing miRNA 132 (miR-132) (11). The lncRNA MCF2L-AS1 plays a critical role in the osteogenic differentiation of bone marrow mesenchymal stem cells, and targeting lncRNA MCF2L-AS1 is a promising strategy to promote osteogenic differentiation (12). LncRNA ZNNT1 acts as a potential tumor suppressor in uveal melanoma by inducing autophagy (13). LncRNA LINK-A modulates growth of breast cancer cells (14). Interestingly, recent studies show involvement of lncRNAs in autoimmune disorders, including RA (15–18); for example, lncRNA LERFS negatively controls rheumatoid synovial aggression and proliferation (19). LncRNA PICSAR promotes cell proliferation, migration, and invasion of RA FLSs (20). However, the role of lncRNAs in the pathogenesis of RA is largely unknown.

In the present study, we determined that lncRNA LINK-A was upregulated in synovial tissues and FLSs from patients with RA. LINK-A regulates inflammation and invasion through HIF-1α mediated by tyrosine protein kinase 6 (PTK6) and leucine-rich repeat kinase 2 (LRRK2) in RA FLSs. We also found that LINK-A promotes aggressive behavior of RA FLSs by sponging miR-1262. Our findings suggest that increased level of LINK-A may contribute to FLS-mediated synovial inflammation and aggression in RA.

## Results

### LncRNA LINK-A has higher-than-normal expression in FLSs and synovial tissues from patients with RA.

**Figure 1:**
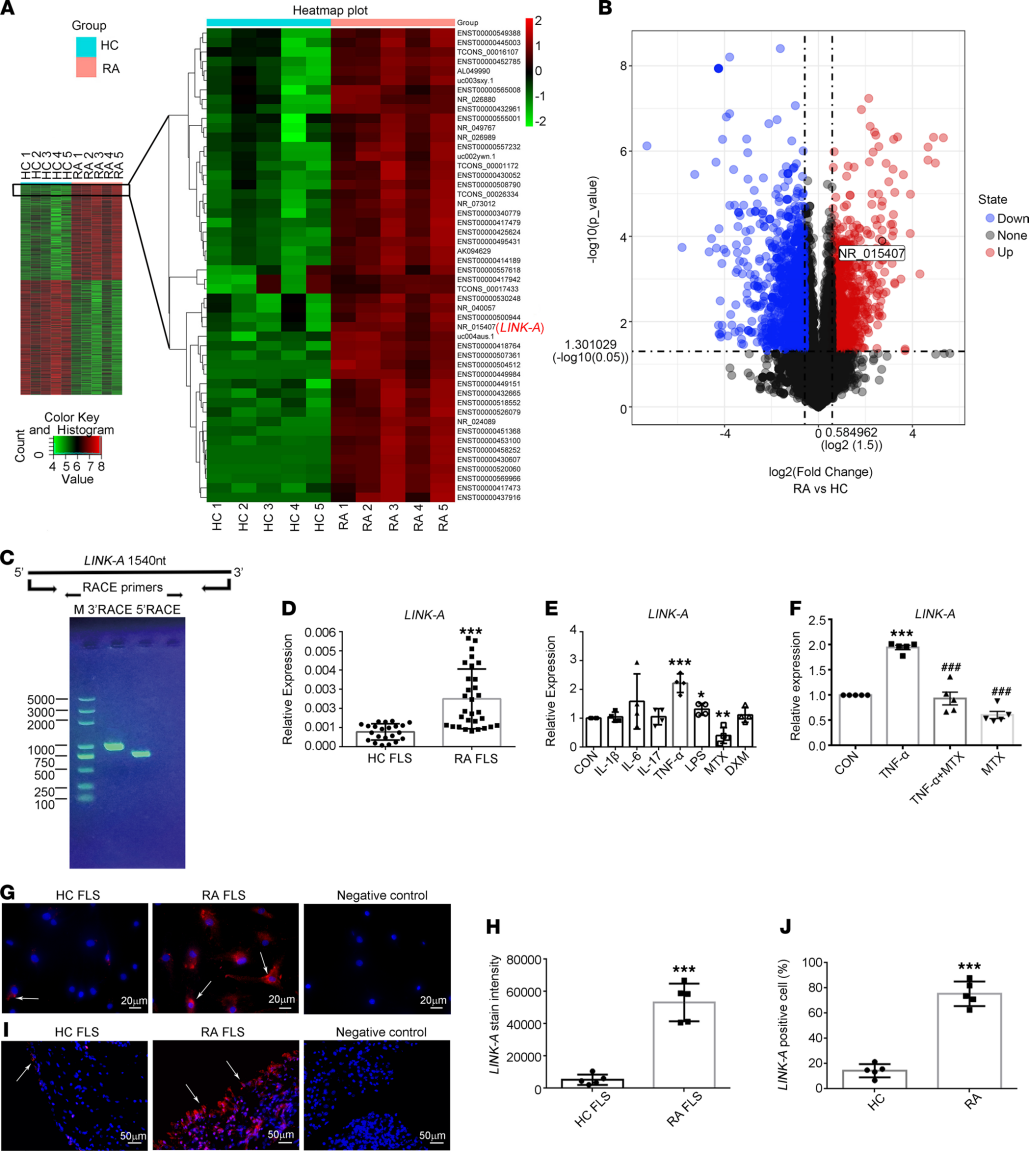
Increased levels of lncRNA LINK-A in FLSs and synovial tissues from patients with RA. (**A**) Total RNA harvested from RA FLSs (*n* = 5) and HC FLSs (*n* = 5) was screened by microarray analysis. Microarray heatmap of distinguishable expression profiles of lncRNAs. (**B**) Volcano plot shows differentially expressed lncRNAs between RA FLSs and HC FLSs. *P* < 0.05, by Student’s *t* test. (**C**) RACE assay of LINK-A. The image shows amplification products of 5′ and 3′ ends of LINK-A. M, marker. (**D**) Verification of LINK-A by RT-qPCR in HC FLSs and RA FLSs. Ct values were normalized to GAPDH. Data are presented as the mean ± SD. (**E**) Expression of LINK-A in RA FLSs treated with IL-1β (10 ng/mL), TNF-α (10 ng/mL), IL-6 (10 ng/mL), IL-17 (10 ng/mL), LPS (10 ng/mL), methotrexate (MTX, 10 μg), and dexamethasone (DXM, 1 μg) for 24 hours. (**F**) Effect of MTX (10 μg) on TNF-α–induced LINK-A expression. (**G** and **H**) Cellular localization of LINK-A was measured by RNA FISH assay. Shown are representative images of LINK-A (red) and nuclei (blue) from 5 different RA patients and HCs. Graph (**H**) shows the quantification of staining intensity for 5 different RA patients and HCs. Original magnification, ×400. (**I** and **J**) LINK-A expression, evaluated by ISH staining, in synovial tissues from HCs and RA patients. Shown are representative images (**I**) and quantification of the percentage of LINK-A–positive cells (**J**) from 5 different RA patients and HCs. A scrambled probe was used as a negative control. White arrows indicate LINK-A–positive (red) cells. Original magnification, ×630. *P < 0.05, **P < 0.01, ***P < 0.001 versus HC FLSs or control (CON); ^###^*P* < 0.001 versus TNF-α, by Student’s 2-tailed *t* test or 1-way ANOVA (for **E** and **F**).

To evaluate the expression pattern of lncRNA in RA, a microarray was used to determine lncRNA expression profiles in FLSs separated from RA patients and healthy controls (HCs). To show that HC FLSs and RA FLSs do cluster separately, principal component analysis (PCA) plots are shown in Supplemental Figure 1; supplemental material available online with this article; https://doi.org/10.1172/jci.insight.146757DS1 Hierarchical clustering analyses showed that the expression differences of 748 lncRNAs were statistically significant (with fold changes of at least 2.0, *P* value of less than 0.05). Among them, 357 were upregulated and 391 were downregulated (Figure 1A, left, and Figure 1B) in the RA group compared with the HC group. We observed that 50 lncRNAs were upregulated in RA FLSs by more than 6-fold (Figure 1A, right).

In the present study, we focused on the lncRNA LINK-A, also known as LINC01139, NR-15407, and LOC339535; neither the sense nor the antisense transcript of it encodes protein (18). Using 5′- and 3′-RACE (rapid amplification of cloned cDNA ends), we confirmed that the full length of LINK-A was 1540 nt (Figure 1C). Quantitative reverse transcription PCR (RT-qPCR) confirmed the significant increase in LINK-A expression in RA FLSs compared with those in HC FLSs (Figure 1D). As inflammatory mediators play important roles in RA, we determined the effects of TNF-α, IL-1β, IL-6, IL-17, and LPS on LINK-A expression in RA FLSs. We found that LINK-A was induced only in treatment with TNF-α or LPS (Figure 1E). We also observed that treatment with MTX, an anchor drug for RA treatment, decreased the expression of LINK-A; however, treatment with DXM, a potent antiinflammatory agent, did not affect LINK-A expression (Figure 1E). MTX treatment also diminished TNF-α–induced LINK-A expression (Figure 1F). Next, we examined the subcellular localization of LINK-A. FISH assay showed that LINK-A was located primarily in the cytoplasm (Figure 1, G and H), which was confirmed by nuclear and cytoplasmic fractionation (Supplemental Figure 2); this prompted us to suspect that LINK-A might perform its biological functions in the cytoplasm. Furthermore, ISH staining confirmed the LINK-A expression was increased in synovial tissue from patients with established RA compared with that in HCs (Figure 1, I and J); moreover, as shown in Supplemental Figure 3, the LINK-A expression in synovial tissue was positively correlated with the severity of synovitis and disease activity score 28 (erythrocyte sedimentation rate) in patients with RA. In addition, we found that LINK-A expression in synovial tissues was not different between RA patients treated with prednisone or disease-modifying antirheumatic drugs and those with no therapy (Supplemental Figure 4). Our data suggest that the increased synovial LINK-A expression may contribute to joint inflammation of RA.

### LncRNA LINK-A positively regulates migration and invasion of RA FLSs.

**Figure 2:**
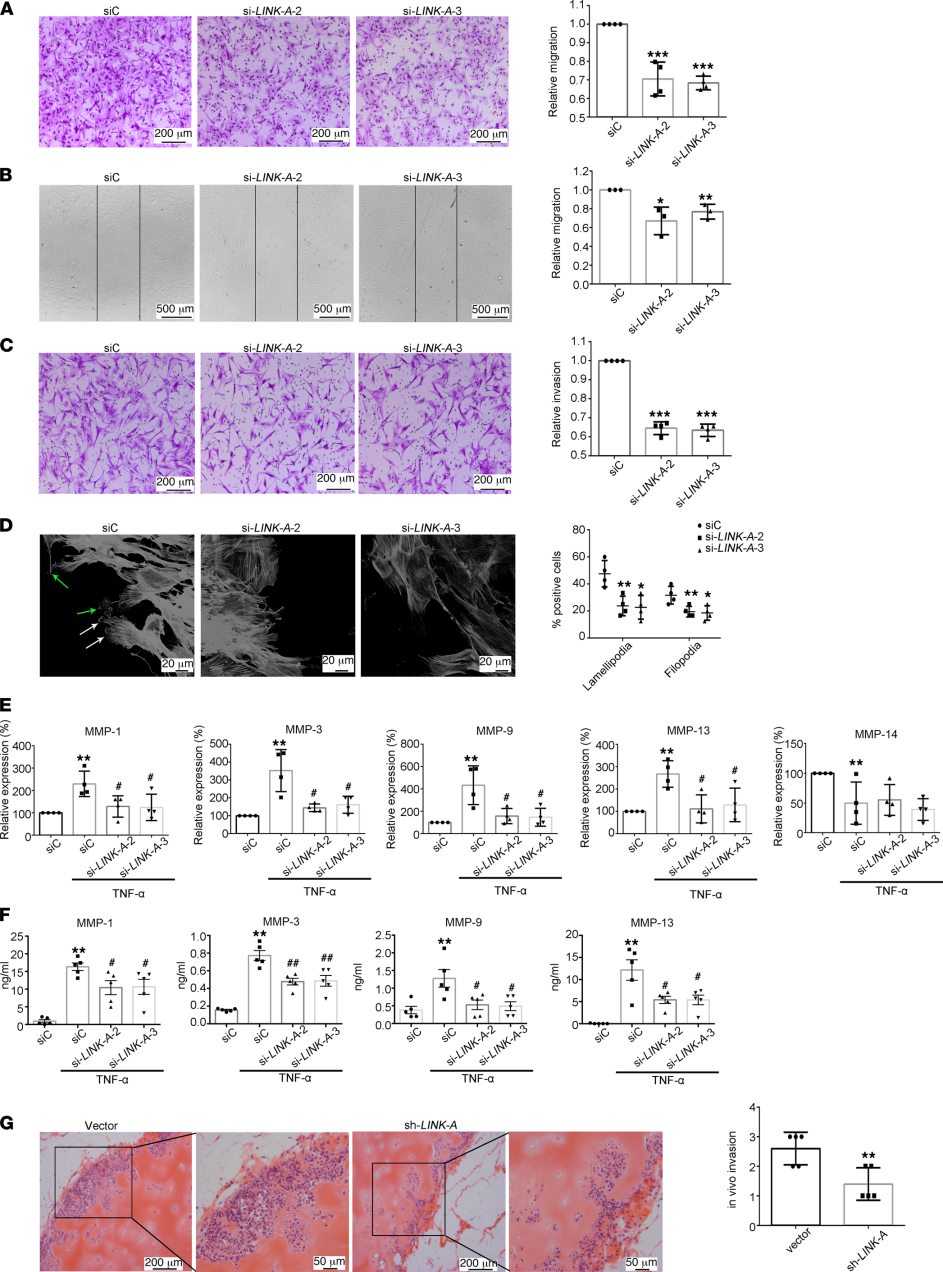
Effects of lncRNA LINK-A knockdown on migration and invasion of RA FLSs. RA FLSs were transfected with LINK-A siRNA (si-LINK-A-2 or si-LINK-A-3) or control siRNA (siC) or LINK-A shRNA (sh-LINK-A) or vector control. (**A**) Chemotaxic migration was measured using a Transwell assay. Representative images (original magnification, ×100) are shown. Graphs show the relative migration rates. (**B**) The cell migration was evaluated using a wound-healing assay. Representative images are shown (original magnification, ×50). The relative migration rate represents the number of migrated cells normalized to the siC. (**C**) In vitro invasion was evaluated using inserts coated with Matrigel Basement Membrane Matrix (BD Biosciences). Representative images (original magnification, ×100) are shown. Graphs show the relative invasion rates. (**D**) Effect of LINK-A knockdown on the pseudopodium formation in RA FLSs. RA FLSs were wounded and then incubated with TNF-α (10 ng/mL) for 4 hours. Representative images are shown (original magnification, ×400). White arrows indicate lamellipodia formation; green arrows indicate filopodia formation. Graph shows the number of RA FLSs with positive lamellipodia or filopodia. (**E** and **F**) Effect of LINK-A knockdown on expression (**E**) and secretion (**F**) of MMPs. MMP expression or secretion was measured by RT-qPCR or ELISA. Data are presented as the mean ± SD. (**G**) Effect of LINK-A shRNA on RA FLS invasion into human cartilage implants transferred under the skin of SCID mice. Arrows show RA FLSs invaded into cartilage. Original magnification, ×200 (left); ×400 right (enlarged). Graph shows the invasion scores. Data are shown as the mean ± SD of 5 independent experiments involving 5 different RA patients. *P < 0.05, **P < 0.01, and ***P < 0.001 versus siC or vector control; ^#^*P* < 0.05 versus TNF-α + siC, by Student’s 2-tailed *t* test (**G**) or 1-way ANOVA (**A**–**F**).

To evaluate the potential role of LINK-A in RA, 3 specific siRNAs for LINK-A (si-LINK-A) were transfected into RA FLSs, respectively. Our data showed that si-LINK-A-2 and si-LINK-A-3 dramatically downregulated the expression of LINK-A in RA FLSs (Supplemental Figure 5); therefore, si-LINK-A-2 and si-LINK-A-3 were used in further experiments. To explore the role of LINK-A in migration, we used the Transwell chamber assay to evaluate the chemotaxic migration of RA FLSs. We found that si-LINK-A transfection decreased FBS-induced migration compared with the control siRNA transfection (Figure 2A). We also used a monolayer wound-scratch assay to evaluate the role of LINK-A in cell migration and found that si-LINK-A treatment reduced the migration of RA FLSs compared with control siRNA (Figure 2B).

The main pathogenic behavior of RA FLSs is to aggressively destruct cartilage and bone. A previous study indicates that the in vitro invasive ability of RA FLSs is related to the rate of joint damage in patients with RA (21). Therefore, we first evaluated the in vitro invasion potential function of LINK-A in RA FLSs using Matrigel-coated Transwells. As shown in Figure 2C, si-LINK-A treatment reduced the invasion compared with control siRNA treatment.

As dynamic reorganization of the actin cytoskeleton plays an essential role in optimal cell migration, we investigated the polymerized actin in FBS-induced RA FLS migration after wounding. As shown in Figure 2D, RA FLSs transfected with control siRNA displayed flat or ruffling lamellipodia and filopodia at their leading edges, while cells transfected with si-LINK-A reduced lamellipodia and filopodia formations. Since Rho GTPases including CDC42, Rac1, and RhoA are critical proteins that control lamellipodia and filopodia, we determined the effect of LINK-A knockdown on the expression and activity of CDC42, Rac1, and RhoA. We found that LINK-A knockdown mitigated the protein expression and activity of RhoA but not Rac1 and CDC42 (Supplemental Figure 6). These data suggest that LINK-A regulates the formation of membrane protrusions in migrating cells by targeting RhoA.

Since MMPs have an important role in rheumatoid joint destruction, we investigated the role of LINK-A in modulating MMPs’ expression. As shown in Figure 2E, transfection with si-LINK-A reduced TNF-α–induced gene expression of MMP-1, MMP-3, MMP-9, and MMP-13. Although MMP-14 plays an important role in regulating FLS invasion (22, 23), we determined that LINK-A knockdown did not influence MMP-14 expression. We also demonstrated that LINK-A knockdown reduced secretion of MMP-1, MMP-3, MMP-9, and MMP-13 (Figure 2F).

Finally, we determined the effect of LINK-A inhibition on in vivo invasion by RA FLSs. We coimplanted RA FLSs transfected with sh-LINK-A or control vector into the left or right flanks, respectively, of SCID mice. RA FLSs transfected with sh-LINK-A exhibited a significant reduction of invasion into cartilage as compared with control vector (Figure 2G).

### LINK-A regulates inflammation but not proliferation and apoptosis in RA FLSs.

**Figure 3:**
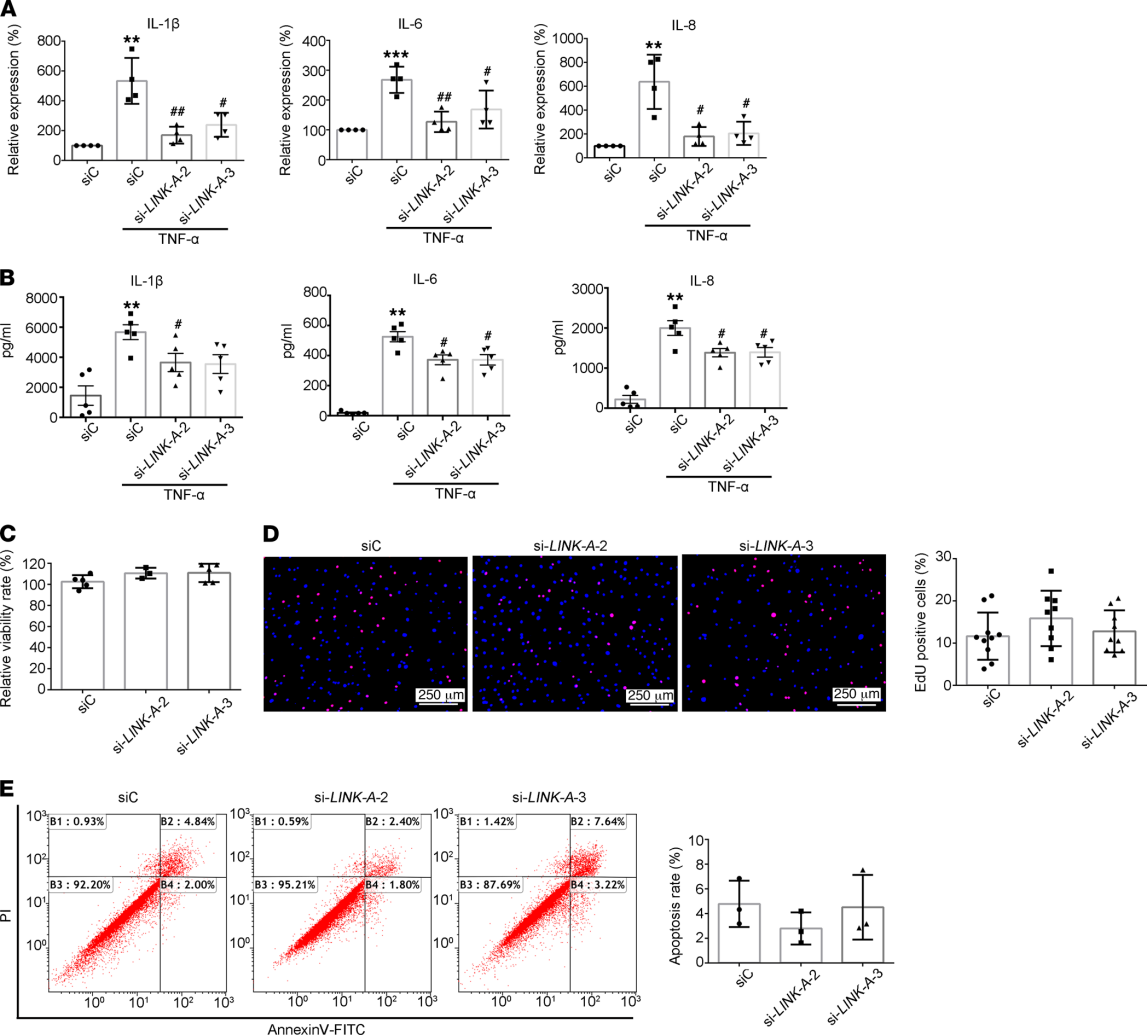
Effects of lncRNA LINK-A knockdown on proinflammatory cytokines, proliferation, and apoptosis of RA FLSs. RA FLSs were transfected with LINK-A siRNA (si-LINK-A-2 or si-LINK-A-3) or control siRNA (siC). (**A**) Effect of LINK-A knockdown on the expression of IL-1β, IL-6, and IL-8. Cytokine expression was detected using RT-qPCR assay. Ct values were normalized to β-actin values. Data are presented as the mean ± SD. (**B**) Effect of LINK-A knockdown on the secretion of IL-1β, IL-6, and IL-8. Cytokine levels were measured using ELISA. Data are presented as the mean ± SD. (**C** and **D**) Effect of LINK-A knockdown on proliferation of RA FLSs. CCK-8 (**C**) or EdU incorporation assay (**D**) was used to evaluate the growth or proliferation. Representative images show proliferation of RA FLSs labeled with EdU (red) and nuclei stained with Hoechst 33342 (blue) (original magnification, ×100). Graphs indicate the mean ± SD of more than 5 independent experiments involving different RA patients. (**E**) Effect of LINK-A knockdown on apoptosis of RA FLSs. The cellular apoptosis rate was evaluated by annexin V and propidium iodide (PI) staining and measured by flow cytometry. Representative flow plots are shown. The apoptosis graph represents the mean ± SD percentage of 3 independent experiments involving different RA patients. *P < 0.05 versus siC, **P < 0.01, and ***P < 0.001 versus siC; ^#^*P* < 0.05, ^##^*P* < 0.01 versus TNF-α + siC, by 1-way ANOVA.

We first determined the role of LINK-A in regulating the expression of proinflammatory cytokines. We observed that si-LINK-A treatment reversed the increase of TNF-α–induced gene expression of IL-1β, IL-6, and IL-8 (Figure 3A). Similarly, we also demonstrated the inhibitory effect of LINK-A knockdown on TNF-α–induced secretion of IL-1β, IL-6, and IL-8 (Figure 3B).

Next, we observed the role of LINK-A in proliferation and apoptosis of RA FLSs. Cell Counting Kit-8 (CCK-8) and EdU assay were used to detect the growth and proliferation, respectively. We found that the cell viability and proliferation rate were not affected in si-LINK-A–treated RA FLSs compared with control siRNA–treated RA FLSs (Figure 3, C and D). Furthermore, we used annexin-FITC staining to detect the apoptosis of RA FLSs by flow cytometry. We observed that the total apoptotic cell number had no difference between RA FLSs treated with si-LINK-A and control siRNA (Figure 3E). These data suggest LINK-A is involved in inflammation but not proliferation and apoptosis in RA FLSs. Collectively, our data suggest that increased LINK-A contributes to synovial inflammation and aggressiveness of RA.

### LINK-A regulates the aggressiveness and inflammation of RA FLSs through PTK6- and LRRK2-mediated HIF-1α.

**Figure 4:**
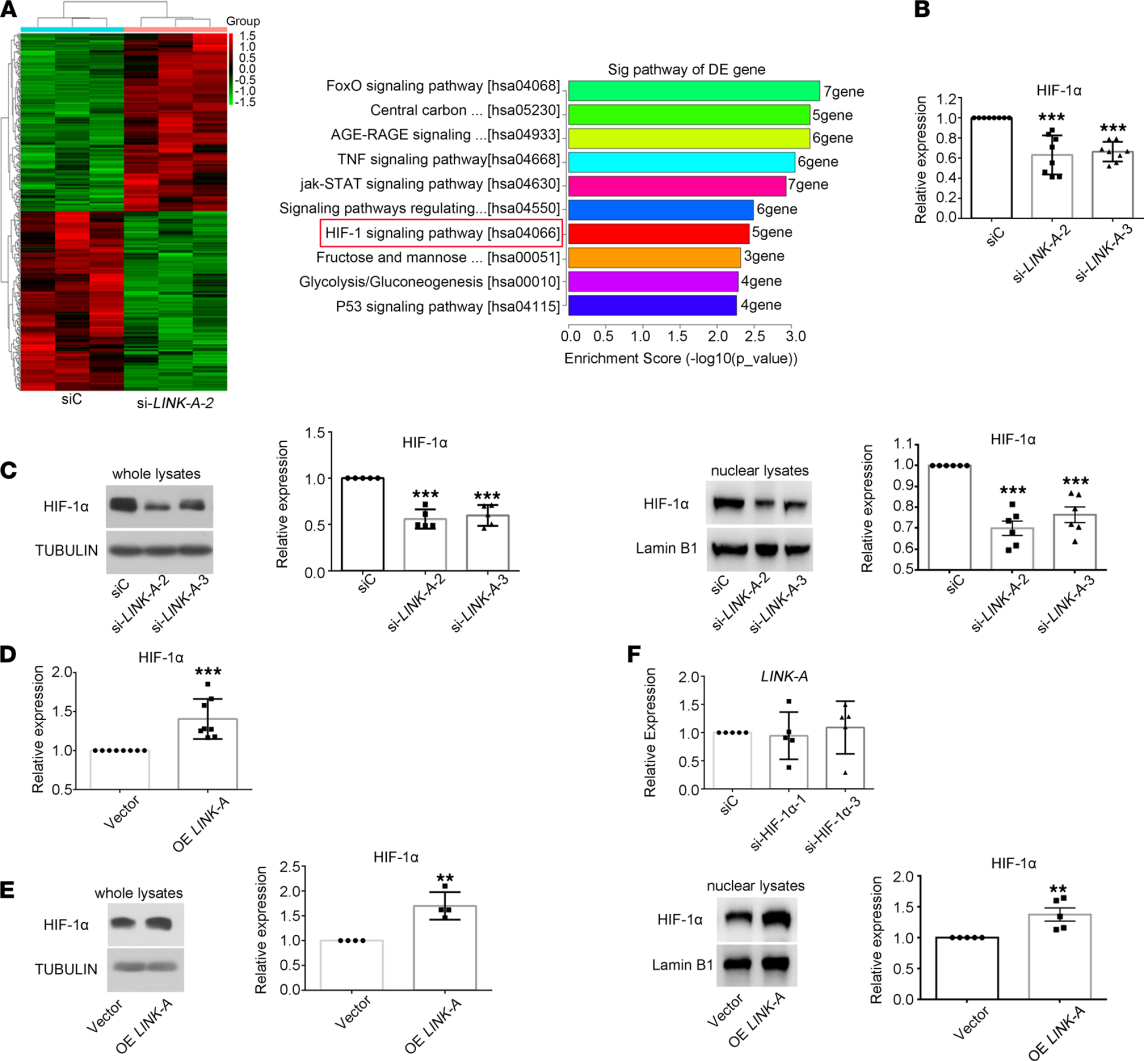
LINK-A functions through HIF-1α in RA FLSs. RA FLSs were transfected with LINK-A siRNA (si-LINK-A-2 or si-LINK-A-3) or HIF-1α siRNA (si-HIF-1α-1 or si-HIF-1α-3) or control siRNA (siC) or were infected with LINK-A–overexpressed lentiviruses (OE LINK-A) or control vector (Vector). The mRNA and protein levels were measured using RT-qPCR and Western blot, respectively. (**A**) Heatmap of distinguishable expression profiles of mRNAs (upper panel) and the KEGG pathway analysis for RNA-Seq (right panel). The bar chart shows the top 10 mRNAs from Kyoto Encyclopedia of Genes and Genomes (KEGG) pathway enrichment analysis. (**B**) Effect of LINK-A knockdown with siRNA on the expression of the mRNA of HIF-1α. (**C**) Effect of LINK-A knockdown on the expression of total and nuclear protein of HIF-1α. (**D**) Effect of LINK-A overexpression on the expression of the mRNA HIF-1α. (**E**) Effect of LINK-A overexpression on the expression of total and nuclear protein of HIF-1α. (**F**) Effect of HIF-1α knockdown on the expression of LINK-A. Data (**C** and **E**, lower or right panel) are expressed as the mean ± SD of densitometry quantification of Western blot from at least 3 independent experiments. **P < 0.01, and ***P < 0.001 versus siC or vector control, by Student’s 2-tailed *t* test (**D** and **E**) or 1-way ANOVA (**B** and **C**).

To explore how LINK-A regulates aggressiveness and inflammation of RA FLSs, we sought to identify intracellular targeting proteins of LINK-A using RNA-Seq. Figure 4A shows the LINK-A silencing-induced top 10 mRNA KEGG pathway enrichment results, including HIF-1 signaling pathway. Consistent with our data, a recent study shows that lncRNA LINK-A activates normoxic HIF-1α signaling in breast cancer tumorigenesis (14); therefore, we speculated that LINK-A might regulate HIF-1α pathway in RA FLSs. As we expected, we demonstrated that LINK-A knockdown with siRNA significantly decreased the mRNA and protein levels of HIF-1α (Figure 4, B and C), while LINK-A overexpression promoted HIF-1α expression (Figure 4, D and E). For exploring the role of LINK-A in regulating HIF-1α activation, we evaluated the effect of LINK-A knockdown or overexpression on nuclear HIF-1α expression. We demonstrated that LINK-A knockdown or overexpression reduced or increased nuclear HIF-1α expression in RA FLSs (Figure 4, C and E). On the other hand, we determined that HIF-1α knockdown did not influence LINK-A expression (Figure 4F). These data suggest that HIF-1α is a downstream targeting protein of LINK-A.

**Figure 5:**
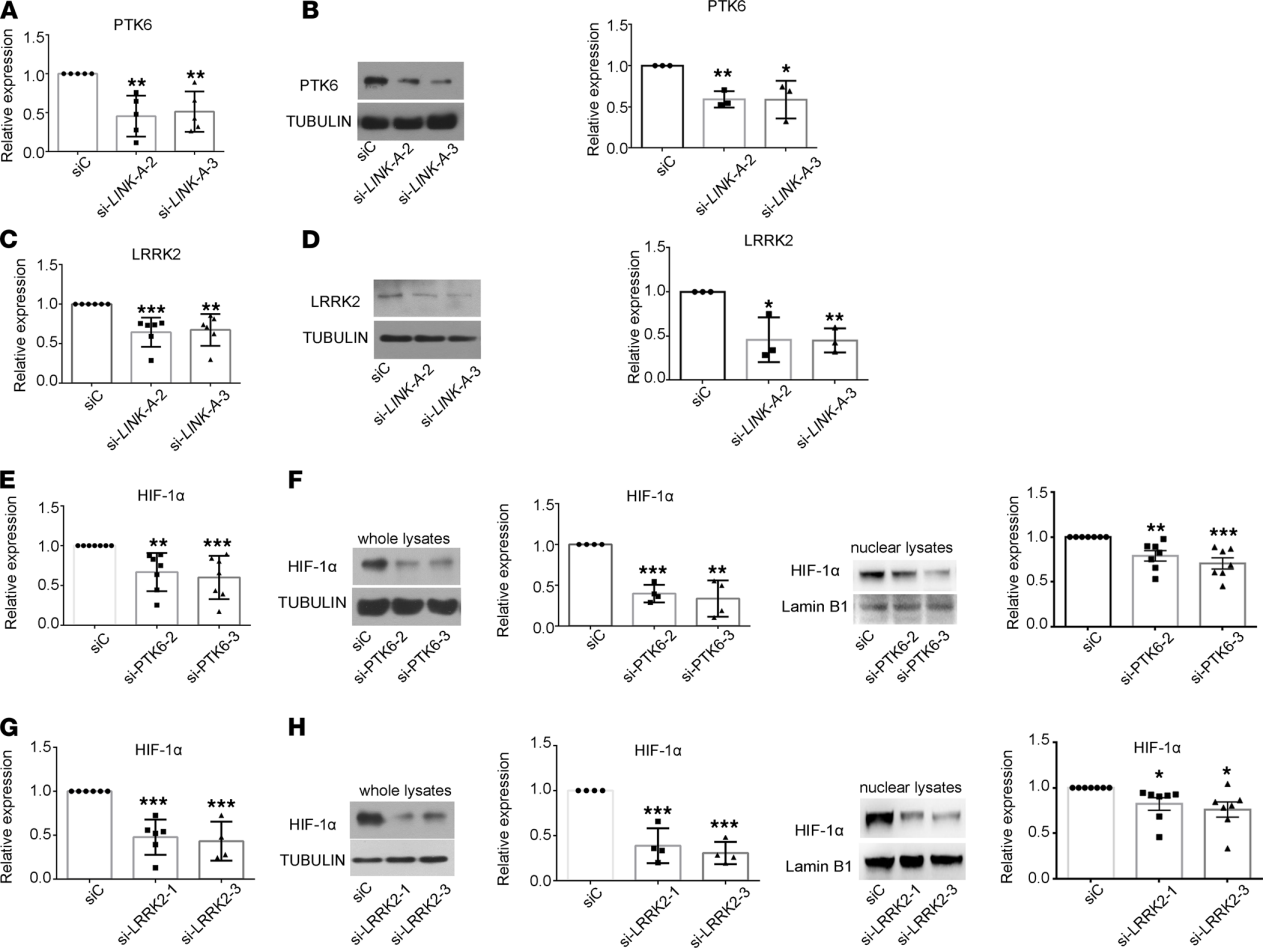
PTK6 and LRRK2 mediate the role of LINK-A in regulating HIF-1α in RA FLSs. RA FLSs were transfected with LINK-A siRNA (si-LINK-A-2 or si-LINK-A-3) or PTK6 siRNA (si-PTK6-2 or si-PTK6-3) or LRRK2 siRNA (si-LRRK2-1 or si-LRRK2-3) or control siRNA (siC). The mRNA and protein levels were measured using RT-qPCR and Western blot, respectively. (**A** and **B**) Effect of LINK-A knockdown with siRNA on the expression of the mRNA (**A**) and protein (**B**) of PTK6. (**C** and **D**) Effect of LINK-A knockdown on the expression of the mRNA (**C**) and protein (**D**) of LRRK2. (**E** and **F**) Effect of PTK6 knockdown with siRNA on the expression of the mRNA (**E**) and protein (**F**) of HIF-1α. (**G** and **H**) Effect of LRRK2 knockdown with siRNA on the expression of the mRNA (**G**) and protein (**H**) of HIF-1α. Data (**B**, **D**, **F**, and **H**, lower or right panel) are expressed as the mean ± SD of densitometry quantification of Western blot from at least 3 independent experiments. *P < 0.05, **P < 0.01, and ***P < 0.001 versus siC, by 1-way ANOVA.

Next, we explored how LINK-A modulates HIF-1α expression. Since it has been shown that LINK-A controls HIF-1α stabilization through interaction with PTK6 and LRRK2 in cancer cells (14), this prompted us to evaluate whether PTK6 and LRRK2 mediate increased LINK-A–induced HIF-1α expression in RA FLSs. We found that LINK-A knockdown decreased the mRNA and protein expression of PTK6 and LRRK2 (Figure 5, A–D); however, knockdown with PTK6 or LRRK2 decreased total and nuclear HIF-1α expression (Figure 5, E–H). Collectively, our data suggest that LINK-A regulates HIF-1α expression and activation via PKT6 and LRRK2 in RA FLSs.

We then investigated the role of HIF-1α in regulating migration, invasion, and inflammation of RA FLSs. We first observed that HIF-1α expression, measured by RT-qPCR, was increased in RA FLSs compared with HC FLSs (Supplemental Figure 7). We used specific siRNAs (si-HIF-1α-1 and si-HIF-1α-3) to inhibit HIF-1α expression (Supplemental Figure 8, A and B). As shown in Supplemental Figure 9, transfection with si-HIF-1α exhibited decreased migration and invasion compared with transfection with control siRNA. Moreover, we found that HIF-1α knockdown decreased expression and secretion of MMP-1, MMP-3, MMP-9, and MMP-13 (Supplemental Figure 10, A and B). HIF-1α knockdown also reduced gene expression and secretion of IL-1β, IL-6, and IL-8 (Supplemental Figure 10, C and D). These data confirm that HIF-1α mediates the role of LINK-A in regulating aggressiveness and inflammation of RA FLSs.

### LINK-A interacts with miR-1262 to regulate the migration and invasion of RA FLSs.

**Figure 6:**
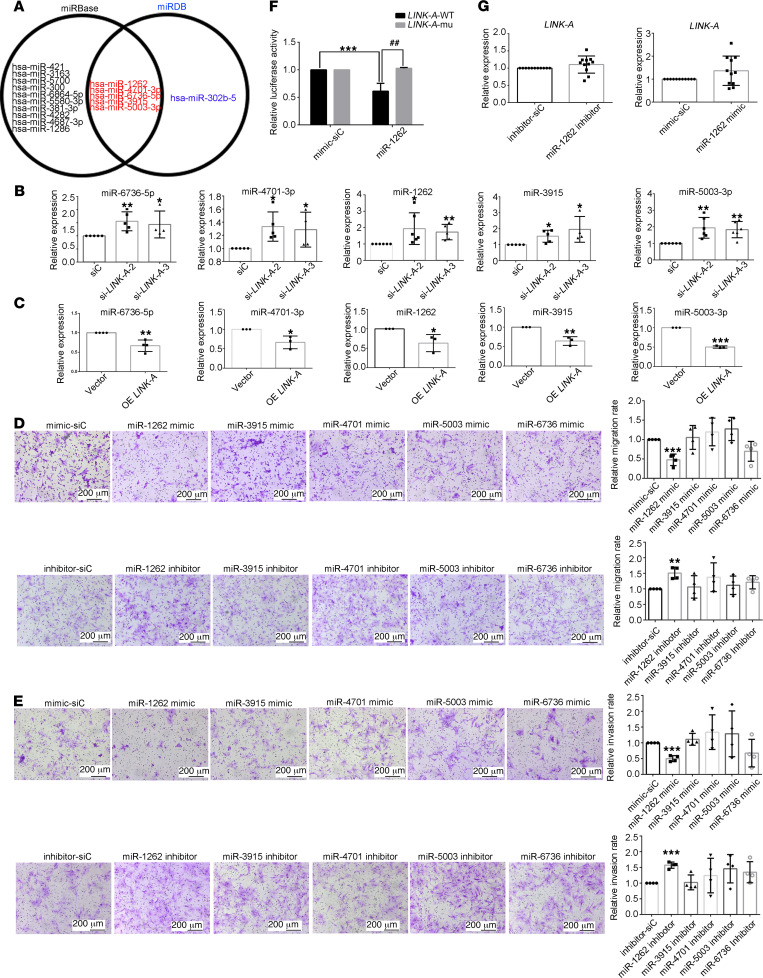
Screening and validation of miRNAs regulated by LINK-A in RA FLSs. RA FLSs were transfected with LINK-A siRNA (si-LINK-A-2 or si-LINK-A-3) or control siRNA (siC) or were infected with LINK-A–overexpressed lentiviruses (OE LINK-A) or control vector (Vector) or treated with miRNA inhibitors or mimics. (**A**) miRBase and miRDB were used to predict miRNAs that can bind to LINK-A. A total of 5 miRNAs, common to both databases, can bind to LINK-A. (**B** and **C**) Effect of LINK-A knockdown (**B**) or overexpression (**C**) on the expression of miRNAs. The miRNAs were detected using RT-qPCR. (**D** and **E**) Effects of miRNA mimics or inhibitors on migration (**D**) and invasion (**E**) of RA FLSs. Migration of RA FLSs was evaluated using a Transwell assay. Invasion was evaluated using inserts coated with Matrigel Basement Membrane Matrix. Representative images (original magnification, ×100) are shown. Graphs show the relative migration or invasion rates. (**F**) Luciferase reporter assay was conducted to verify the targeting effect of miR-1262 on LINK-A sequence. (**G**) Effect of miR-1262 inhibition or mimics on LINK-A expression. Data are presented as the mean ± SD from at least 3 independent experiments. *P < 0.05, **P < 0.01, ***P < 0.001; ^##^*P* < 0.01 versus siC or vector or normal control (NC), by Student’s 2-tailed *t* test (**C** and **G**) or 1-way ANOVA (**B**, **D**, **E**, and **F**).

Since it is mainly located in cytoplasm, in addition to interaction with cytoplasm proteins, LINK-A has a possibility of interaction with miRNAs and works as competing endogenous RNAs (ceRNAs) to promote miRNA exhaustion, thereby regulating gene expression at the posttranscriptional level (24, 25). Therefore, to verify whether LINK-A acts as ceRNAs for a certain miRNA, we used miRBase and miRDB to predict the potential miRNA binding with LINK-A. The analysis showed that a total of 5 miRNAs, including miR-6736-5p, miR-4701-3p, miR-1262, miR-3915, and miR-5003-3p, had potential to bind LINK-A (Figure 6A). We further demonstrated that LINK-A knockdown by siRNA increased expression of miR-6736-5p, miR-4701-3p, miR-1262, miR-3915, and miR-5003-3p; however, LINK-A overexpression decreased the expression of these miRNAs (Figure 6, B and C). This confirmed the interaction of cytoplasmal LINK-A with miRNA in RA FLSs. Next, we determined the role of these miRNAs in regulating RA FLS biological functions. We observed that only transfection of miR-1262 mimics reduced the migration and invasion of RA FLS, and treatment with miR-1262 inhibitor increased migration and invasion of RA FLSs (Figure 6, D and E). However, we found that treatment with mimics or inhibitors of miR-1262, miR-6736-5p, miR-4701-3p, miR-3915, and miR-5003-3p did not affect the expression of proinflammatory factors and MMPs in RA FLSs (Supplemental Figure 11). In addition, treatment with mimics or inhibitor of miR-1262 did not influence the secretion of proinflammatory factors and MMPs (Supplemental Figure 12). Moreover, we also found that treatment with mimics or inhibitor of miR-1262 did not influence the proliferation of RA FLSs (Supplemental Figure 13). Moreover, we observed increased levels of miR-1262 in RA FLSs compared with HC FLSs (Supplemental Figure 14). These data suggest that miR-1262 may mediate the role of LINK-A in regulating migration and invasion of RA FLSs.

Finally, to determine whether LINK-A binds directly with miR-1262, luciferase reporter assays were conducted. As shown in Figure 6F, LINK-A-WT and miR-1262 significantly reduced the luciferase activities, but the LINK-A-MUT and miR-1262 had little influence on the luciferase activity. Furthermore, we demonstrated that miR-1262 inhibition or mimics did not influence LINK-A expression (Figure 6G). This suggests that miR-1262 was the directly targeting miRNA of LINK-A.

**Figure 7:**
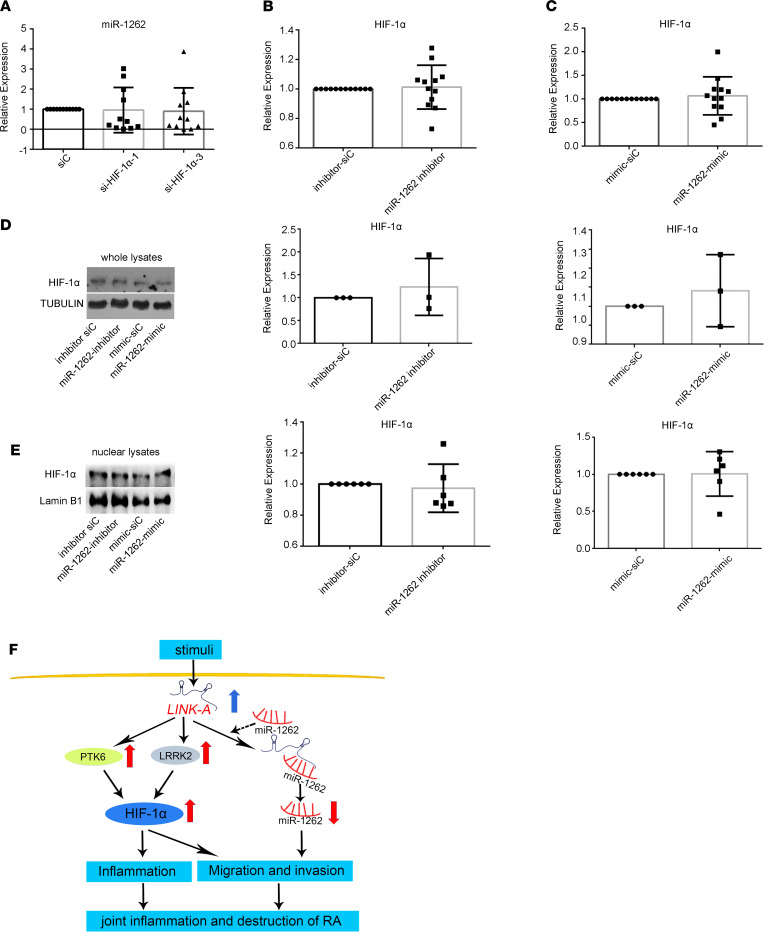
The relationship between miR-1262 and HIF-1α in RA FLSs. RA FLSs were treated with miRNA inhibitors or mimics or transfected with HIF-1α siRNA (si-HIF-1α-1 or si-HIF-1α-3). The miRNAs and mRNA were detected using RT-qPCR. The protein levels were measured by Western blot. (**A**) Effect of HIF-1α knockdown on the expression of miR-1262. (**B** and **C**) Effect of miR-1262 inhibitor (**B**) or mimics (**C**) on mRNA expression of HIF-1α. (**D**) Effect of miR-1262 inhibitor or mimics on protein expression of total HIF-1α. (**E**) Effect of miR-1262 inhibitor or mimics on protein expression of nuclear HIF-1α. Data (**A**–**C**) are presented as the mean ± SD from at least 3 independent experiments. Graphs (**D** and **E**, right panel) are expressed as the mean ± SD of densitometry quantification of Western blot results from at least 3 independent experiments. (**F**) Proposed model for LINK-A–mediated regulation of the synovial migration, invasion, and inflammation in RA FLSs.

Since our above results show that HIF-1α mediates the role of LINK-A in regulating RA FLS functions, we observed the relationship between miR-1262 and HIF-1α. We found that HIF-1α knockdown did not affect miR-1262 expression in RA FLSs (Figure 7A). Moreover, we showed that miR-1262 inhibitor or mimics did not influence the gene expression of HIF-1α (Figure 7, B and C). We also demonstrated that miR-1262 inhibitor or mimics did not affect the expression of total and nuclear protein of HIF-1α (Figure 7, D and E), suggesting that LINK-A promotes RA FLS migration and invasion by sponging miR-1262 in an independent HIF-1α pathway.

## Discussion

In the present study, we found increased expression of lncRNA LINK-A in FLSs and synovial tissues from patients with RA. LncRNA LINK-A functions as a positive regulator of migration, invasion, and inflammation in RA FLSs. Mechanistically, we identified that LINK-A regulates RA FLS functions through 2 independent pathways. One is through a PKT6- and LRRK2-mediated HIF-1α pathway to modulate aggressiveness and inflammation of RA FLSs; the other is a sponging interaction with miR-1262 to regulate RA FLS aggressiveness (Figure 7F). Our data suggest that increased synovial LINK-A contributes to joint inflammation and destruction of RA.

Recent studies show some lncRNAs are involved in regulation of RA FLS functions (17–19, 26); however, the contribution of lncRNAs to the pathogenesis of RA is largely unknown. In this work, we observed increased expression levels of lncRNA LINK-A in FLSs and synovial tissues from patients with RA. LINK-A knockdown decreased migration, invasion, and proinflammatory cytokine secretion of RA FLSs. These data indicate that LINK-A positively regulates the FLS aggressiveness and inflammation and that the increase in LINK-A in RA FLSs might contribute to increased rheumatoid synovial inflammation and aggression, leading to joint destruction. In line with our findings, recent reports show that LINK-A modulates migration and invasion of osteosarcoma cell lines (27) and ovarian carcinoma cells (28). Although previous studies indicate that LINK-A is associated with proliferation and apoptosis in tumor cells (29) and podocytes (30), we found that LINK-A knockdown did not affect proliferation and apoptosis of RA FLSs, suggesting that LINK-A may possess a cell type–specific function in modulating cellular proliferation and apoptosis.

We further explored the underlying mechanism(s) by which LINK-A regulates the aggressiveness and inflammation of RA FLSs. LINK-A is located predominantly in the cytoplasm, which indicates that it might function by interacting with other cytoplasmic factors. A recent study shows that LINK-A controls HIF-1α stabilization and activation of HIF-1α transcriptional programs under normoxic conditions in triple-negative breast cancer (14). In our study, the KEGG pathway analysis of RNA-Seq data also revealed that LINK-A inhibition affects the HIF-1 signaling pathway.

We further demonstrated that HIF-1α is a downstream targeting protein of LINK-A in RA FLSs. On the other hand, it has been indicated that HIF-1α plays an important role in regulating RA FLS functions. For instance, HIF-1α is involved in regulating proinflammatory pathways and invasive behavior in RA FLSs (31–33). HIF-1α also modulates rheumatoid synovial bioenergetics, including glycolytic metabolism (34, 35), inflammatory cell recruitment, and angiogenesis (36). In addition, HIF-1α promotes the interactions between RA FLSs and synovial T cells and B cells (37). Here, we also observed the inhibitory effect of HIF-1α knockdown on the migration, invasion, and expression of proinflammatory cytokines and MMPs in RA FLSs. These findings suggest that HIF-1α mediates the role of LINK-A in regulating RA FLS invasion and inflammation.

Next, we explored interactions between LINK-A and HIF-1α in RA FLSs. It has been shown that LINK-A interacts with PKT6 and LRRK2 and thereby promotes HIF-1α phosphorylation and protein stabilization under normoxic conditions in breast cancer cells (14). In this work, we demonstrated that LINK-A knockdown decreased the expression of PTK6 and LRRK2, while PTK6 or LRRK2 inhibition by siRNA decreased HIF-1α protein expression in RA FLSs. Furthermore, PTK6 or LRRK2 knockdown decreased migration, invasion, and inflammation of RA FLSs (data not shown). In line with our findings, PTK6 promotes cancer cell aggressiveness (38, 39). LRRK2 is also involved in inflammatory response and cell migration (40, 41). Taken together, our data suggest that increased LINK-A promotes PTK6- and LRRK2-mediated HIF-1α protein expression and subsequently regulates aggressiveness and inflammation of RA FLSs. However, we do not eliminate the possibility that other signaling pathways mediate the role of LINK-A in controlling RA FLS functions.

Increasing evidence indicates that lncRNAs can be competitively binding to miRNAs and work as ceRNAs (42). For instance, lncRNA PICSAR plays an important role in promoting rheumatoid synovial invasion and joint destruction by sponging miR-4701-5p (20). LncRNA GAPLINC controls tumor-like biological behaviors of RA FLSs by miRNA sponging (26). Thus, we determined whether LINK-A interacts with miRNA using molecular biological analysis. In our study, miR-1262 was selected as a superior target of LINK-A according to its significantly increased expression after LINK-A inhibition and its role in regulating RA FLS function. Our subsequent analyses showed that miR-1262 inhibition or mimics did not affect LINK-A expression, and the targeting relationship between miR-1262 and LINK-A was confirmed using luciferase reporter assay. Furthermore, we observed that miR-1262 inhibition or mimics increased or reduced RA FLS migration and invasion but did not affect the expression of proinflammatory cytokines, suggesting miR-1262 is only involved in aggressive action of RA FLSs. Consistent with our findings, one research group has shown that miR-1262 regulates invasion of breast cancer cells (43). These data suggest that miR-1262 might be a new target for controlling synovial aggression and joint destruction of RA. However, it needs to be further explored how miR-1262 modulates RA FLS aggression in the future. Collectively, in this study, we propose a mechanism in which LINK-A acts as a miR-1262 sponge to regulate aggressive behavior of RA FLSs.

In summary, we have described the regulatory function of lncRNA LINK-A in the migration, invasion, and inflammation of FLSs through 2 independent pathways; one is dependent on HIF-1α, and the other is dependent on miR-1262. Our study suggests that increased expression of lncRNA LINK-A in FLSs may contribute to the synovial aggression and inflammation that characterize rheumatoid joint abnormalities.

## Methods

### Reagents and antibodies.

Recombinant human IL-1β, IL-6, IL-17α, and TNF-α were obtained from R&D Systems (Bio-Techne), and LPS was purchased from MilliporeSigma. DMEM, FBS, antibiotics, trypsin EDTA, PBS, and other products for cell culture were obtained from Invitrogen, Thermo Fisher Scientific. The primary antibodies used were as follows: anti–HIF-1α (catalog number 36169), anti-LRRK2 (catalog number 5559), anti-CDC42 (catalog number 2466), and anti-RhoA (catalog number 2117) were purchased from Cell Signaling Technology. Anti–alpha Tubulin (catalog number ab7291) and anti-PTK6 (catalog number ab233392) were purchased from Abcam. Anti-Rac1 (catalog number 05-389) was purchased from MilliporeSigma. Anti–Lamin B (catalog number 66095-1-Ig) was obtained from Proteintech.

### Preparation of human synovial tissues and FLSs.

Synovial tissues were obtained from active RA patients (25 women and 5 men, 35–68 years old) who were undergoing synovectomy of knee joint or total knee replacement. RA was diagnosed according to the 2010 American College of Rheumatology/European League Against Rheumatism classification criteria (44). The demographics of patients with RA are shown in Supplemental Table 1. We obtained HC synovial tissues from 22 people (18 women and 4 men, 31–57 years old) who underwent a traumatic single above-knee amputation and had no history of acute or chronic arthritis. RA synovitis was scored as follows (45): 0–1: no synovitis; 2–3: slight synovitis; 4–6: moderate synovitis; and 7–9: strong synovitis. The scores were calculated in a blinded fashion by 2 pathologists.

Synovial tissues were cut into small pieces and digested with collagenase (1 mg/mL) for 3 hours at 37°C to separate FLSs. FLSs were cultured in DMEM/F12 with 10% FBS at 5% CO_2_ and 37°C. In the present study, we used cells from passages 4–6, during which time they were a homogeneous population of cells (1% CD11b positive, 1% phagocytic, and 1% FcgRII and FcgRIII receptor positive).

### Microarray and data analysis.

Total RNA was prepared using TRIzol (MilliporeSigma) from HC FLSs (*n* = 5) and RA FLSs (*n* = 5) and quantified by NanoDrop ND-1000 (Thermo Fisher Scientific). RNA integrity was evaluated by standard denaturing agarose gel electrophoresis. After elimination of ribosomal RNA (rRNA) using the mRNA-ONLY Eukaryotic mRNA Isolation Kit (Epicentre), the samples were amplified and transcribed into fluorescent complementary RNA (cRNA) along the entire length of the transcripts without 3′ bias, using a random priming method. The labeled cRNAs were hybridized onto the Human lncRNA Array v2.0 (8 × 60K, Arraystar). After hybridization and washing, the arrays were scanned using the Agilent Scanner G2505B (Agilent Technologies), and Agilent Feature Extraction software v10.7.3.1 (Agilent Technologies) was used to analyze the acquired array images. Quantile normalization and subsequent data processing were performed by GeneSpring GX v11.5.1 software (Agilent Technologies). We evaluated differentially expressed lncRNAs with statistical significance through volcano plot filtering and calculated a *P* value by Student’s *t* test. The threshold set for up- and downregulated genes was a fold change more than 2.0 and *P* value less than 0.05. Heatmaps representing differentially regulated genes were produced using Cluster v3.0 software. We finally used hierarchical clustering to show the differential lncRNAs’ expression pattern among the samples. The microarray data discussed in this article were deposited in the National Center for Biotechnology Information’s (NCBI) Gene Expression Omnibus (GEO) database (GEO GSE181614; https://www.ncbi.nlm.nih.gov/geo/query/acc.cgi?acc=GSE181614).

### Rapid amplification of cloned complementary DNA.

SMARTer RACE cDNA Amplification Kit (Clontech) was used for rapid amplification of 5′ and 3′ ends of LINK-A. The primer sequences for 5′ and 3′ RACE are listed in Supplemental Table 2. We performed RACE according to the manufacturer’s instructions.

### Fluorescence in situ hybridization.

We performed FISH following the established protocol as described previously (46). FLSs were briefly rinsed in PBS and fixed in 4% formaldehyde in PBS (pH 7.4) for 15 minutes at room temperature. The cells were then permeabilized in freshly made 0.5 % v/v Triton X-100 in PBS containing 2 mM voriconazole on ice for 10 minutes. After being washed with 2 × saline-sodium citrate, the cells were hybridized with RNA FISH Probes (125 μM, Biosearch Technologies) at 37°C for 16 hours in a moist chamber. After incubation with RNA FISH wash buffer A for 30 minutes, the cells were counterstained with DAPI. Then, the cells were incubated with RNA FISH wash buffer B for 2 minutes and imaged by a confocal laser scanning microscope (LSM710; Carl Zeiss) equipped with LSM ZEN 2008 software.

### In situ hybridization.

To evaluate the expression pattern of LINK-A in synovial tissues, ISH was performed with RNA FISH Probe (Biosearch Technologies). Briefly, after deparaffinization and rehydration, the sections were digested with proteinase K (15 μg/mL) for 10 minutes at 37°C and subsequently dehydrated. Then the sections were hybridized with RNA FISH Probe (125 μM) at 37°C for 16 hours. After incubation with RNA FISH wash buffer A for 30 minutes, they were counterstained with DAPI for 30 minutes at 37°C. Finally, the sections were incubated with RNA FISH wash buffer B for 5 minutes, then imaged by a confocal laser scanning microscope (LSM710; Carl Zeiss) equipped with LSM ZEN 2008 software.

After being washed and blocked, the sections were incubated at room temperature for 1 hour with RNA FISH Probes. Finally, the sections were stained with nitroblue tetrazolium/5-Bromo-4-chloro-3-indolyl phosphate (Roche Life Science), counterstained with nuclear fast red, and mounted.

### Quantitative real-time PCR.

Total RNA was obtained using the Takara PrimeScript RT Reagent Kit (Takara Bio) according to the manufacturer’s instructions, and RT-qPCR was performed using the Bio-Rad CFX96 system (Bio-Rad Laboratories). The primers employed for real-time PCR are listed in Supplemental Table 3. To quantify the relative expression of each gene, we normalized Ct values to the endogenous reference (*Δ*Ct = Ct target — Ct GAPDH) and compared them using a calibrator and the *ΔΔ*Ct method (*ΔΔ*Ct = *Δ*Ct sample — *Δ*Ct calibrator). All experiments were performed in triplicate.

### Cell nuclear protein extraction.

Nuclear protein was extracted using NE-PER Nuclear and Cytoplasmic Extraction Reagents kit (Thermo Fisher Scientific). RA FLSs were harvested to microcentrifuge tubes and pelleted by centrifugation at 500*g* for 2–3 minutes. The supernatant was carefully removed, and the cell pellet was dried and then suspended. Ice-cold CER II was added to the tube. The tube was vortexed and incubated, then centrifuged for 5 minutes at maximum speed in a microcentrifuge (~16,000*g*). The insoluble (pellet) fraction, which contains nuclei, was suspended in ice-cold Nuclear Extraction Reagent. We continued vortexing it for 15 seconds every 10 minutes, for a total of 40 minutes. The tube was centrifuged at maximum speed (~16,000*g*) in a microcentrifuge for 10 minutes. Finally, the supernatant (nuclear extract) fraction was harvested to a clean, prechilled tube.

### Western blot.

Protein concentrations were measured using the Bicinchoninic Acid Protein Assay Kit (Thermo Fisher Scientific). Equal amounts of protein were solubilized in Laemmli buffer (62.5 mM Tris-HCl pH 6.8, 10% glycerol, 2% SDS, 5% β-mercaptoethanol, and 0.00625% bromophenol blue), boiled for 5 minutes, separated by SDS-PAGE, and transferred to nitrocellulose membranes. The membranes were probed with indicated primary antibodies in TBS/Tween 20 containing 5% nonfat milk at 4°C overnight. Then the membranes were incubated with the secondary antibodies (anti–rabbit IgG, 7074, Cell Signaling Technology) for 1 hour at room temperature. Enhanced chemiluminescence (ECL; GE Healthcare Life) was used to visualize immunoreactive bands. Each blot was representative of at least 3 similar independent experiments.

### Infection of overexpression lentivirus.

LINK-A overexpression lentivirus was purchased from Genechem; we used empty vector lentivirus expressing GFP as NCs only. Cells were cultured in 6-well plates until 60% confluent and infected with lentivirus particles in the presence of 10 g/mL polybrene at a MOI of 30. The cells were cultured for at least 3 days before further experiments.

### Transfection of siRNA.

The LINK-A, HIF-1α, PTK6, and LRRK2 siRNAs and nonsilence control siRNAs were purchased from RiboBio. The target sequences of siRNAs are listed in Supplemental Table 4. The cells were seeded in 6-well plates at 60%–70% confluence and transiently transfected with the above siRNAs (100 nM) or the corresponding control siRNA using Lipofectamine 3000 (Thermo Fisher Scientific). Experiments were performed 48 hours after transfection.

### Chemotaxis migration and invasion in vitro.

Chemotaxis migration of FLSs was performed using the Boyden chamber method with a filter 6.5 mm in diameter and at a pore size of 8.0 μm (Transwell; Corning Labware Products). Briefly, DMEM containing 10% FBS was put in the lower wells, and suspended FLSs (6 **×** 10^4^ cells/mL) in serum-free DMEM were put in the upper wells. The plate was incubated at 37°C in 5% CO_2_ for 12 hours. Then the nonmigrating cells were removed from the filter’s upper surface using a cotton swab, and the filters were fixed in methanol for 15 minutes and stained with 0.1% crystal violet for 15 minutes. The chemotaxis was quantified using an optical microscope to count the stained cells that had migrated to the lower side of the filter. The stained cells were counted as the mean number of cells per 10 random fields for each assay. For the measurement of invasion in vitro, similar experiments were conducted using inserts coated with BD Matrigel Basement Membrane Matrix (BD Biosciences).

### Wounding migration.

RA FLSs were covered to 70% confluence on 35 mm culture dishes, then were serum starved for 12 hours and subsequently wounded using 200 μL pipette tips. The culture dishes were washed 3 times with PBS to remove detached cells, and the remaining cells were grown in DMEM containing 10% FBS for 48 hours. The migration was quantified by counting the cells that had moved beyond a reference line.

### Evaluation of in vivo invasion of RA FLSs into human cartilage implants.

Cultured FLSs were suspended in sterile saline solution to a final volume of 60 mL for each sponge before implantation. The normal human cartilage, obtained from nonarthritic patients undergoing knee surgery for traumatic injuries, was cut into 5 to 8 mm^3^ pieces. For implantation, 2 implants containing cartilage and the same population of RA FLSs were inserted under the skin at the left flank of 4-week-old female SCID mice (Guangdong Medical Laboratory Animal Center, Guangzhou, China).

The mice were raised and housed under standard conditions with air filtration (20 ± 2°C; 12 hours light/12 hours dark). Briefly, on the day of implantation, normal human cartilage was cut into pieces. A piece of cartilage (5–8 mm^3^) was inserted with a cube of insert sponge (80 mm^3^). The sponge was soaked with 4 × 10^5^ FLSs suspended in sterile saline. Three pieces of sponge containing FLSs and cartilage were inserted into the skin of the anesthetized mouse under sterile conditions. After 50 days of implantation, the implants were detached from the sacrificed mice and embedded immediately, snap-frozen, and stored at –70°C until further experiments. The explant sections were stained with H&E, and each specimen was examined for the grade of invasion of FLSs into cartilage as described previously (47). The aggression level was scored as follows: 0 = no or minimal invasion; 1 = visible invasion (2-cell depth); 2 = invasion (5-cell depth); and 3 = deep invasion (more than 10-cell depth). Two experienced scientists evaluated the sections in a blinded manner.

### EdU proliferation assays.

FLSs were cultured in 96-well plates in complete media until 80%–90% confluent, then were incubated with 50 μM EdU in complete medium for 6 hours. We used the Cell-Light EdU DNA Cell Proliferation Kit (RiboBio) to detect cell proliferation according to the manufacturer’s instructions.

### Cell Counting Kit-8.

Cell viability was assessed using CCK-8. Briefly, the indicated cells (5 × 10^3^/well) were plated in a 96-well plate and incubated for 24 hours. After lentivirus infection, the cells were incubated with CCK-8 reagent (100 μL/mL medium) for 2 hours at 37°C, and the absorbance of the solution was read at 450 nm using a microplate reader (Varioskan Flash, Thermo Fisher Scientific).

### Apoptosis assays.

FLS apoptosis assay was performed using staining with PI and FITC annexin V (both from BD Biosciences) according to the manufacturer’s instructions. In brief, the cells were suspended in 1× binding buffer at a concentration of 1 × 10^6^ cells/mL. Then, the cell suspension (100 μL) was transferred to a 1.5 mL Eppendorf tube, mixed with 5 μL annexin V and 5 μL PI, and incubated for 20 minutes at room temperature in darkness. The samples were analyzed using flow cytometry within 1 hour.

### Measurement of secretion of MMPs and proinflammatory cytokines.

The levels of MMP-1, MMP-3, MMP-9, MMP-13, IL-1β, IL-6, and IL-8 were measured by ELISA according to the manufacturer’s instructions (Cusabio).

### RNA-Seq and bioinformatics analysis.

Total RNA was quantified using a NanoDrop ND-1000 instrument, and random primers were used to produce cDNA. In our experiments, all RNA integrity number values were more than 7, and 260:280 ratios measured by NanoDrop were 1.0–2.0. The sequencing library was constructed according to the following steps: first, total RNA was enriched by oligo(dT) magnetic beads (rRNA was eliminated); and second, the RNA-Seq library was prepared using KAPA Stranded RNA-Seq Library Prep Kit (Illumina), which incorporates dUTP into the second cDNA strand and renders the RNA-Seq library strand specific. The completed libraries were confirmed with an Agilent Technologies 2100 Bioanalyzer and quantified by an absolute-quantification qPCR method. For sequencing the libraries, the barcoded libraries were mixed, denatured to single-stranded DNA in NaOH, captured on an Illumina flow cell, amplified in situ, and subsequently sequenced for 150 cycles from both ends on an Illumina HiSeq 4000 instrument.

Raw sequencing data, produced from Illumina HiSeq 4000 that passed the Illumina purity filter, were used for the following analysis. Trimmed reads (trimmed 5′, 3′ adaptor bases) were aligned to the reference genome. Based on statistical analysis of the alignment (mapping ratio, rRNA/mtRNA content, fragment sequence bias), we evaluated whether the results could be used for further data analysis. If so, we further evaluated the expression profiling, differentially expressed genes, and differentially expressed transcripts. Hierarchical clustering, Gene Ontology, PCA, correlation analysis, and pathway analysis were performed for the differentially expressed genes in R or Python software for statistical computing and graphics. The RNA-Seq data discussed in this article were deposited in the NCBI’s GEO database (GSE181615; https://www.ncbi.nlm.nih.gov/geo/query/acc.cgi?acc=GSE181615).

### Measurement of RhoA, Rac1, and CDC42 activity.

RA FLSs were cultured for 24 hours at a density of 1 × 10^5^ cells/well in 35 mm culture dishes in serum-free medium. A G-LISA RhoA, Rac1, and CDC42 Activation Assay Kit (Cytoskeleton) was used to detect RhoA, Rac1, and CDC42 activity according to the manufacturer’s recommendations.

### Statistics.

The data are expressed as the means ± SD. Presented values were derived from at least 5 independent experiments for the in vitro experiments. We performed the experimental procedures and treatment and data analyses with blinding. To reduce baseline variability between independent experiments, we normalized the quantitative analysis of immunoblots and mRNA expression. The data were normalized as the fold over the mean of the control. We compared 2 groups by Student’s 2-tailed *t* test; 3 or more different groups were evaluated by 1-way ANOVA. *P* value less than 0.05 was considered significant. We performed statistical analyses of the data using SPSS v13.0 software.

### Study approval.

Our study was performed according to the recommendations of the Declaration of Helsinki and approved by the Medical Ethical Committee of the First Affiliated Hospital, Sun Yat-sen University, China. All patients gave written informed consent to participate in the study. Animal experiments were approved by Animal Care and Ethics Committee of Sun Yat-sen University.

## Author contributions

JW, CS, and RL performed the majority of the experiments and analyzed and interpreted the data. CW, YX, ML, and YK collected clinical samples and analyzed and interpreted the data. SX and MS performed the in vivo experiments. JW, LL, XC, and HX contributed to the study concept and design. JW, LL, and HX drafted the manuscript. XC, LL, and HX contributed to study supervision.

## Supplementary Material

Supplemental data

## Version 1 12/08/2021

Electronic publication
