# Supplementary material for: Increased long noncoding RNA LINK-A contributes to rheumatoid synovial inflammation and aggression
Source: JCI Insight. 2021 Dec 8;6(23):e146757. doi: 10.1172/jci.insight.146757 (PMC8675191; doi:10.1172/jci.insight.146757)
Supplement: Supplemental data [file jciinsight-6-146757-s086.pdf]

Supplemental data

Fig. S1 **Principal component analysis (PCA) plots for lncRNA expression cluster of HC FLS and RA FLS.**

Fig. S2 Localization of LINK-A by nuclear/cytoplasm fractionation. RT-qPCR analysis of cytoplasmic and nuclear fraction of HC FLSs revealed that LINK-A expression was enriched in cytoplasm. Values are expressed relative to expression level of nucleus. GAPDH served as a cytoplasmic marker; U6 served as a nuclear marker.

Fig. S3 Correlation of synovial LINK-A expression with the synovitis score (A) and DAS28-ESR (B) in RA patients. LINK-A expression and synovitis score of RA patients were evaluated by ISH and H&E staining, respectively. Correlation analysis was performed by Spearman's rank order correlation test.

Fig. S4 The quantification of the percentage of LINK-A-positive cells in synovial tissues from RA patients treated with prednisone or MTX (GC+MTX) and those with no therapy (naive). Data are shown as the mean ± SD.

Fig. S5 Efficiency of LINK-A knockdown. RA FLS were transfected with specific LINK-A siRNA1-3 for 48 h, and silencing efficiency was evaluated by RT-qPCR. Data show the silencing efficiency of LINK-A siRNA from 3 independent experiments. Ct values are normalized to β-actin. *****P* < 0.001 vs. control siRNA (siC), by 1-way ANOVA

Fig. S6 Effect of LINK-A knockdown on the expression and activity of RhoA, Rac1 and CDC42. RA FLS were transfected with LINK-A siRNA 2 or LINK-A siRNA 3 for 48 h. (A) Effect of LINK-A knockdown on the expression of RhoA, Rac1 and CDC42. The protein expression was measured by Western blot. Data (lower panel) are expressed as the mean ± SD of densitometry quantification of Western blot from at least 6 independent experiments (B) Effect of LINK-A knockdown on the activity of CDC42, Rac1 and RhoA. The activity of RhoA, Rac1 and CDC42 was detected using G-LISA. Data are expressed as the mean ± SD from at least 6 independent experiments. **P* < 0.05 vs. control siRNA (siC), by 1-way ANOVA

Fig. S7 Expression of HIF-1α in FLS from RA patients and healthy control (HC) subjects. HIF-1α expression was determined by RT-qPCR. Data are presented as the mean ± SD

from at least 3 independent experiments. **P < 0.01 vs. HC FLS, by Student's t test

Fig. S8 Efficiency of HIF-1α knockdown. RA FLS were transfected with HIF-1α siRNA 1-3 for 48 h and subjected to RT-qPCR analysis (**A**) and Western blot (**B**). A representative blot of at least 3 independent experiments is shown. The data shown are the mean ± SD from at least 3 independent experiments. ****P < 0.001 vs. siC, by 1-way ANOVA

Fig. S9 Effect of HIF-1α knockdown on migration, invasion in RA FLSs. RA FLS were transfected with siRNA-2 and siRNA-3 for HIF-1α (si HIF-1α-2, si HIF-1α-3) or siC. Migration (**A**) and invasion (**B**) were evaluated using a Boyden chamber. An invasion assay was performed using inserts coated with a Matrigel basement membrane matrix. The migrated or invaded FLSs were stained violet using a Diff-Quick kit (left panel, original magnification, 100 ×). The migration or invasion index represents the number of migrated or invaded cells normalized to siC-treated cells. Data show the mean ± SD for samples from 4 different RA patients. ****P < 0.001 vs. siC, by 1-way ANOVA

Fig. S10 Effect of HIF-1α knockdown on expression and secretion of MMPs and pro-inflammatory cytokines. RA FLS were transfected with siRNA-2 and siRNA-3 for HIF-1α (si HIF-1α-2, si HIF-1α-3) or siC with or without TNF-α stimulation for 24 hours. The mRNA expression of MMP and cytokine was detected using RT-qPCR assay. Ct values were normalized to β-actin values. MMP and cytokine secretion was measured using ELISA. (**A** and **B**) Effect of HIF-1α knockdown on expression and secretion of MMPs. (**C** and **D**) Effect of HIF-1α knockdown on expression and secretion of proinflammatory cytokines. Data are presented as the mean ± SD. *P < 0.05, **P < 0.01 vs. siC, $^{\#}P$ < 0.05, $^{\#\#}P$ < 0.01 vs. siC + TNF-α, by 1-way ANOVA

Fig. S11 Effect of miRNAs inhibition or mimics on the expression of MMPs (A and C) and pro-inflammatory cytokines (B and D) in RA FLSs. RA FLS were treated with miRNAs inhibitors or mimics with or without TNF-α stimulation for 24 hours. MMP and pro-inflammatory cytokine expression was measured by RT-qPCR. Ct values were normalized to β-actin values. Data are presented as the mean ± SD. *P < 0.05, **P < 0.01 vs. siC, by 1-way ANOVA

Fig. S12 Effect of miRNA1262 inhibition or mimics on secretion of MMPs (A and B) and pro-inflammatory cytokines (C and D) in RA FLSs. RA FLSs were treated with

miRNA 1262 (miR-1262) mimics or inhibitors for 24 hours. MMPs and cytokines secretion was measured using ELISA. Data are presented as the mean ± SD. **P* < 0.05, ***P* < 0.01 vs. siC, by 1-way ANOVA

Fig. S13 Effect of miRNA1262 inhibition or mimics on the proliferation of RA FLSs. RA FLSs were treated with miRNA 1262 (miR-1262) inhibitors (A) or mimics (B) for 24 hours. EdU incorporation assay (**D**) was used to evaluate the proliferation. Representative images show proliferation of RA FLSs labeled with EdU (red) and nuclei stained with Hoechst 33342 (blue) (original magnification, 100 ×). Graphs (lower panels) indicate the mean ± SD of 8 independent experiments involving 8 different RA patients.

Fig. S14 Decreased levels of miRNA 1262 (miR-1262) in FLS from patients with RA. Expression of miR-1262 was measured using RT-qPCR assay. Ct values were normalized to β-actin values. Data are presented as the mean ± SD. **P* < 0.05 vs. HC FLS, by Student's *t* test

Fig.S1

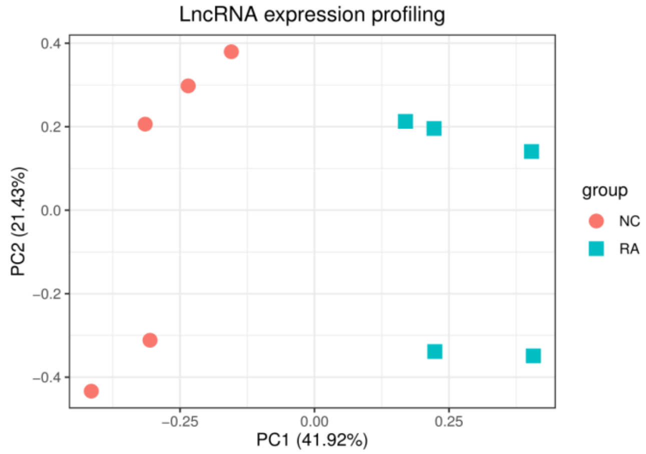

Fig.S2

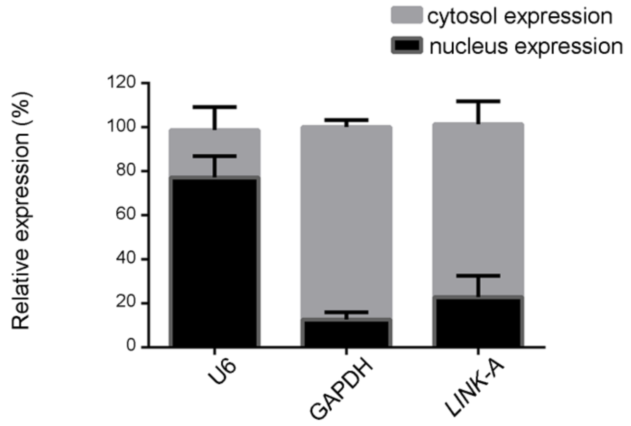

Fig.S3

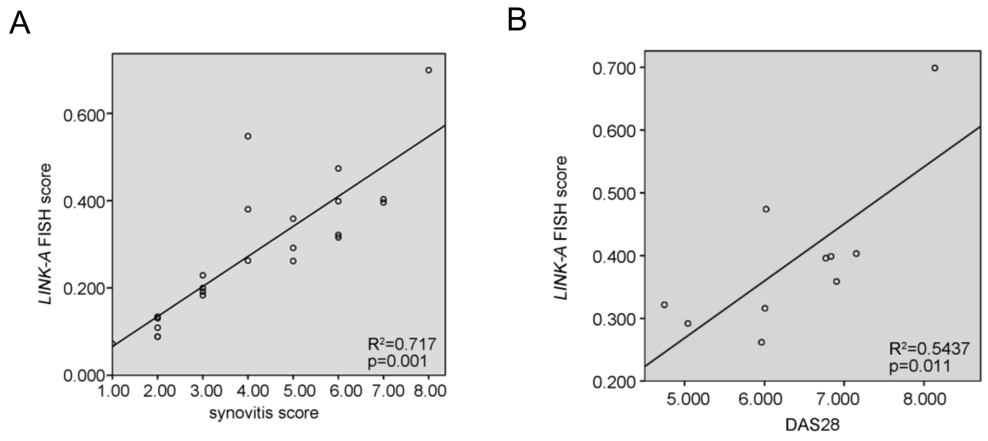

Fig.S4

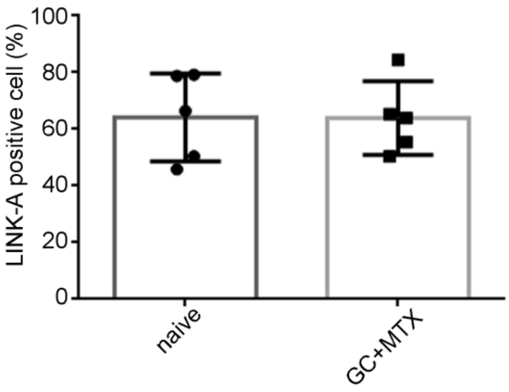

Fig.S5

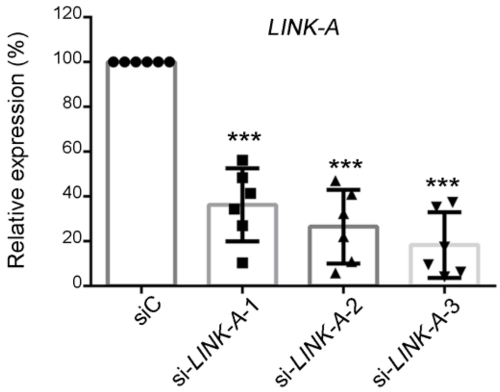

Fig.S6

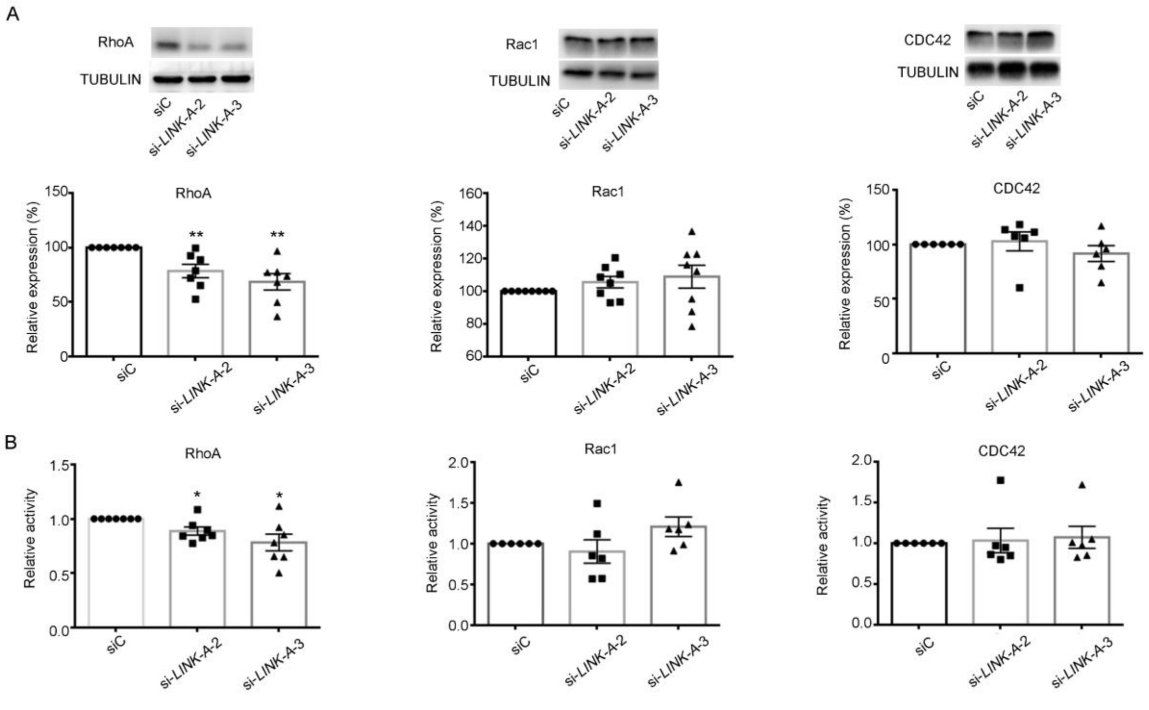

Fig.S7

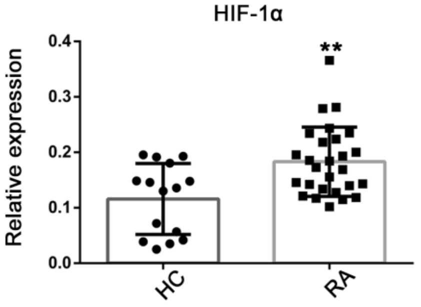

Fig.S8

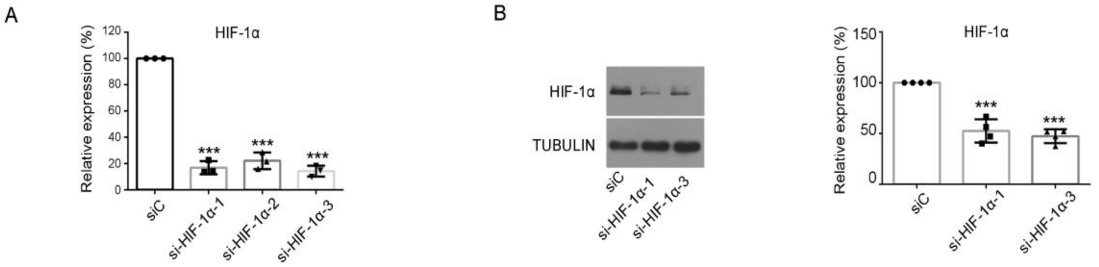

Fig.S9

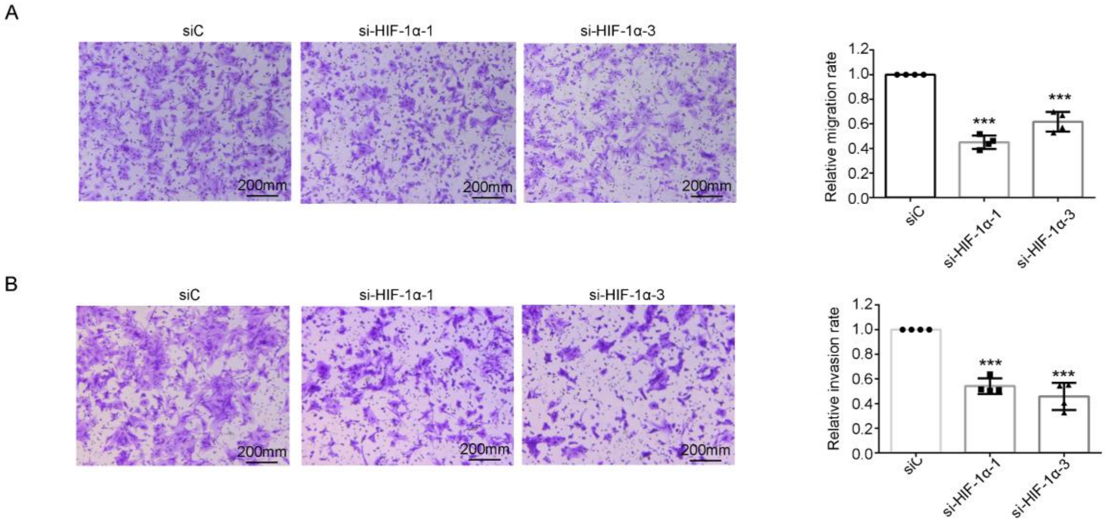

Fig.S10

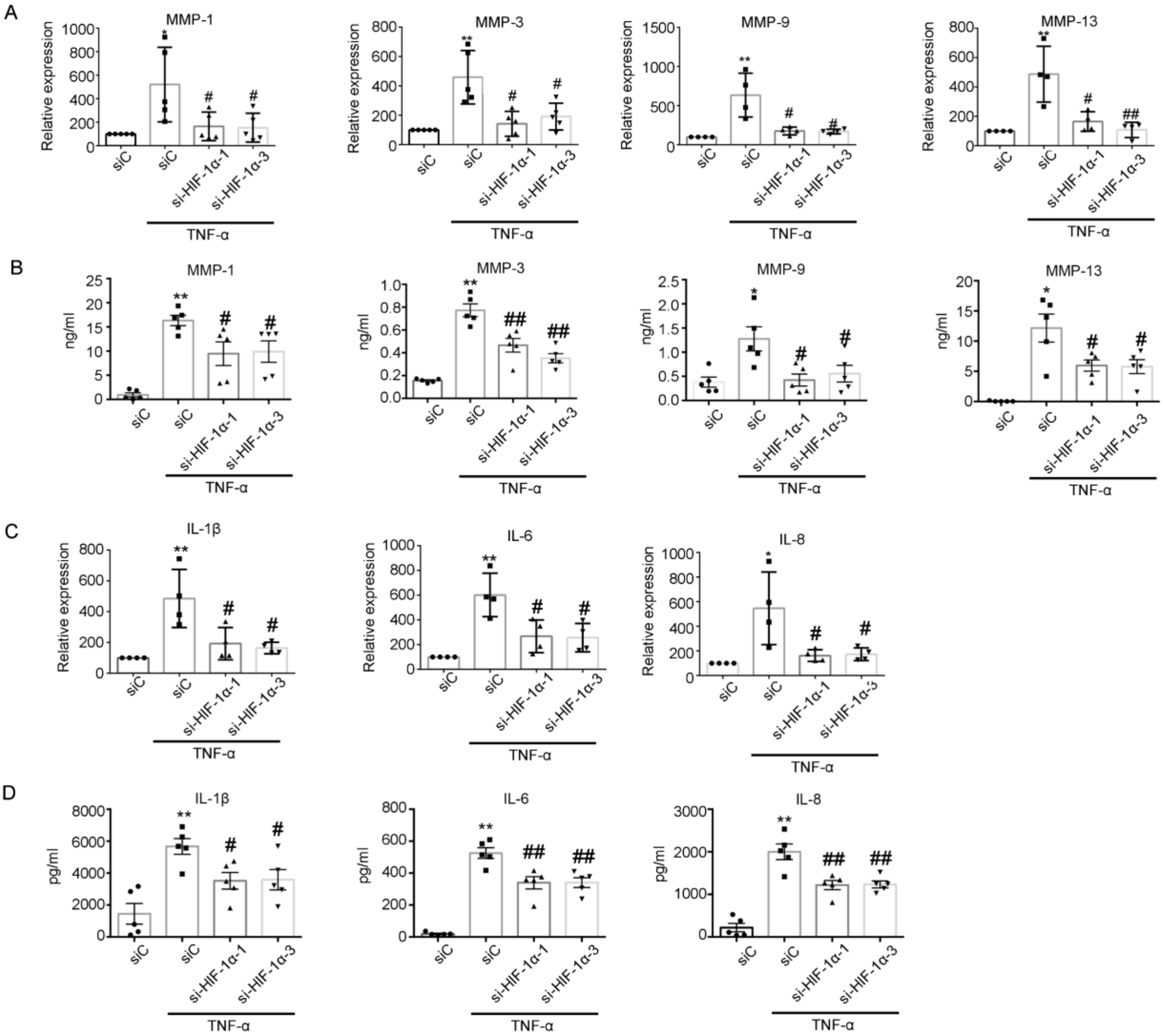
A
MMP-1
MMP-3
MMP-9
MMP-13
Relative expression
siC
si-HIF-1α-1
si-HIF-1α-3
TNF-α
B
ng/ml
C
IL-1β
IL-6
IL-8
D
pg/ml

Fig.S11

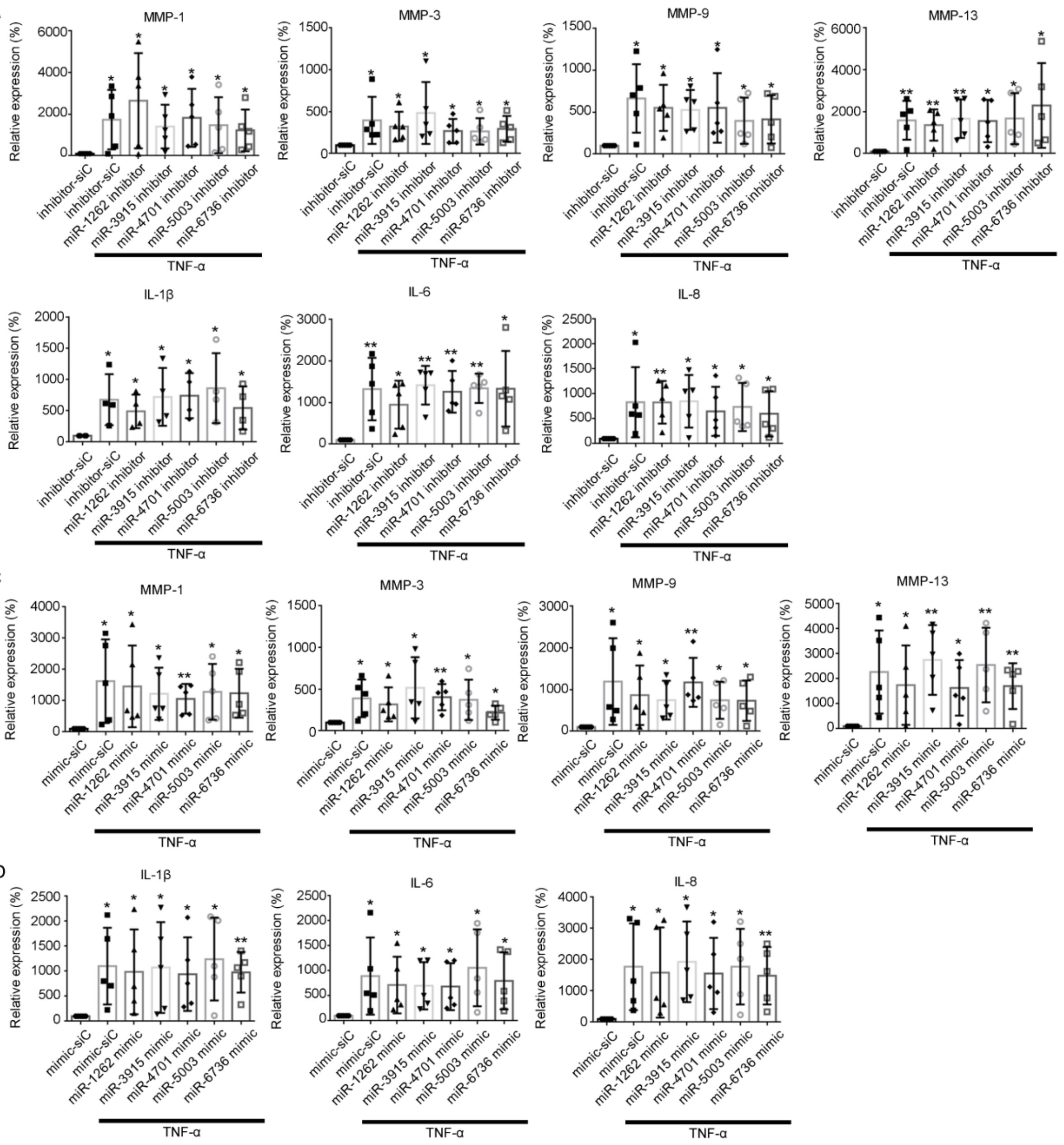
A
MMP-1
MMP-3
MMP-9
MMP-13
B
IL-1β
IL-6
IL-8
C
MMP-1
MMP-3
MMP-9
MMP-13
D
IL-1β
IL-6
IL-8
Relative expression (%)
inhibitor-siC
inhibitor-siC
miR-1262 inhibitor
miR-3915 inhibitor
miR-4701 inhibitor
miR-5003 inhibitor
miR-6736 inhibitor
mimic-siC
mimic-siC
miR-1262 mimic
miR-3915 mimic
miR-4701 mimic
miR-5003 mimic
miR-6736 mimic
TNF-α

Fig.S12

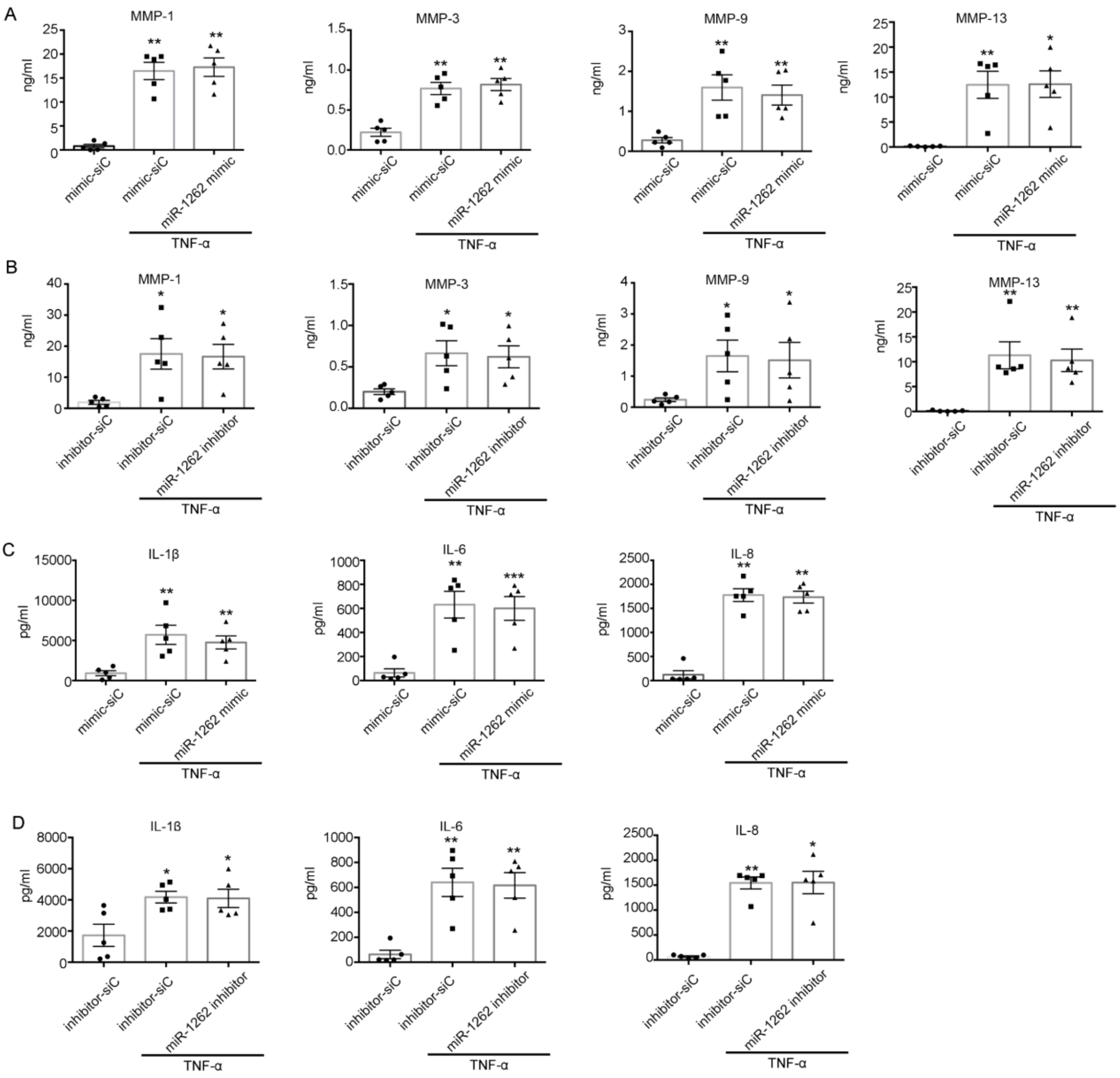
A
MMP-1
ng/ml
mimic-siC
mimic-siC
miR-1262 mimic
TNF-α
MMP-3
MMP-9
MMP-13
B
inhibitor-siC
inhibitor-siC
miR-1262 inhibitor
C
IL-1β
IL-6
IL-8
pg/ml
D
IL-1ß

Fig.S13

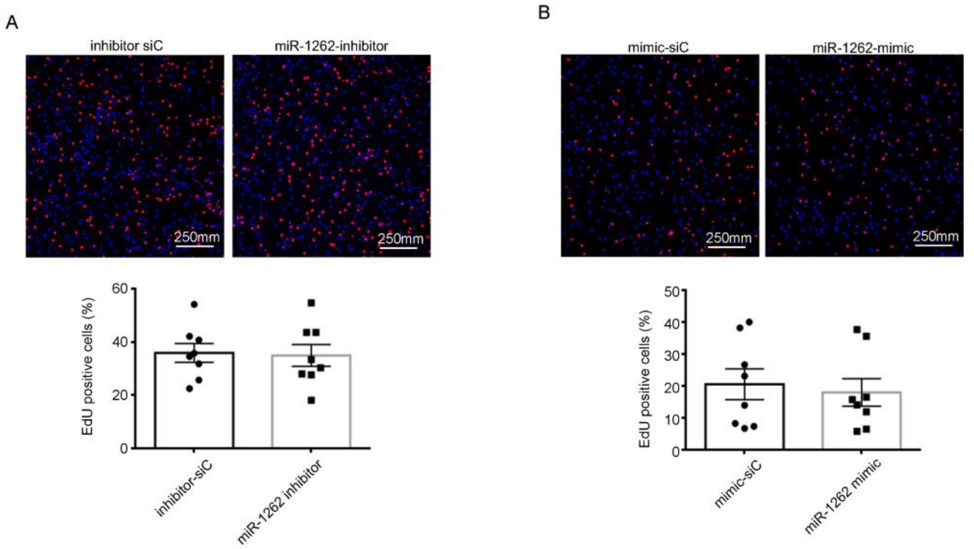

Fig.S14

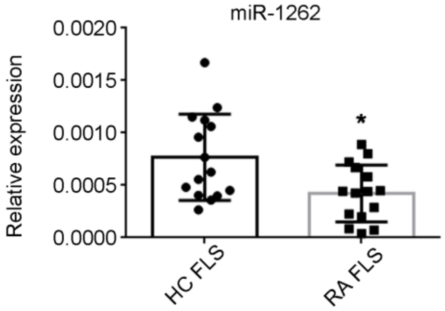

Tables

Table S1. Demographic and clinical features of patients with active RA.

Age, yrs (mean ± SD)	57.6±9.6
Female, n (%)	25（83.3）
Male, n (%)	5 (16.7)
Disease duration, yrs (mean±SD)	5.7±6.8
Rheumatoid factor-positive, n (%)	25（83.3）
Anti-CCP-positive, n (%)	16 (53.3)
DAS28 (CRP) (mean±SD)	5.2 ± 1.4
Previous medications, n (%)	
Prednisone (<10mg/d)	13 (43.3)
Methotrexate	7 (23.3)
Leflunomide	3(10.0)
Sulfasalazine	1 (3.3)
Hydroxychloroquine	3 (10.0)

Table S2 Sequences of RACE primers

LINK-A 5' RACE	GCAGAGCATCCCTGTTTCCC
LINK-*A* 3' RACE	GCTAAAGGCATCTTTGTCCG
UPM	CTAATACGACTCACTATAGGGCAAGCAGTGGTATCAACGCAGAGT

Table S3 Sequences of RT-PCR primers

Name	Forward	Reverse
β-actin	TCAAGATCATTGCTCCTCCTGAG	ACATCTGCTGGAAGGTGGACA
GAPDH	GCACCGTCAAGGCTGAGAAC	TGGTGAAGACGCCAGTGGA
LINK-A	TTCCCCCATTTTTCCTTTTC	CTCTGGTTGGGTGACTGGTT
HIF-1α	ATCCATGTGACCATGAGGAAATG	TCGGCTAGTTAGGGTACACTTC
PTK6	ACCTGGAGTCGCAGAATTACA	GCCTGGCTAACCCGAAGTC
LRRK2	CCTGTTGTGGAAGTGTGGGA	TCTCTTTCTGCTTTTGTGTACCT
MMP-1	CTCTGGAGTAATGTCACACCTCT	TGTTGGTCCACCTTTCATCTTC
MMP-3	TGTAAAGAAACCTTCCTGCAA	TTTAAAACACAGTATGCCCAA
MMP-9	TGTACCGCTATGGTTACACTCG	GGCAGGGACAGTTGCTTCT
MMP-13	TCCTGATGTGGGTGAATACAATG	GCCATCGTGAAGTCTGGTAAAAT
MMP-14	CATCTGTGACGGGAACTTTGA	GGCAGTGTTGATGGACGCA
IL-1β	ATGATGGCTTATTACAGTGGCAA	GTCGGAGATTCGTAGCTGGA
IL-6	ACTCACCTCTTCAGAACGAATTG	CCATCTTTGGAAGGTTCAGGTTG
IL-8	ACTGAGAGTGATTGAGAGTGGAC	AACCCTCTGCACCCAGTTTTC

hsa-miR-1262	ATGGGTGAATTTGTAGAAGGAT
hsa-miR-3915	TTGAGGAAAAGATGGTCTTATT
hsa-miR-4701-3p	TGGGTGATGGGTGTGGTGT
hsa-miR-5003-3p	TACTTTTCTAGGTTGTTGGGG
hsa-miR-6736-5p	GGGTGAGGGCATCTGTGGT

Table S4 Sequences of siRNA

siRNA: *LINK-A*	1	TGTCTAAGGTGGAGATTAC
	2	AGATGTAGTTCTAGTTCAT
	3	GGTCTTCATTCTTACGCTT
siRNA ： HIF-1α	1	GGAACATGATGGTTCACTT
	2	CTACCCACATACATAAAGA
	3	CCAGCAACTTGAGGAAGTA
siRNA ： PTK6	1	CCGAGCTTGTGAACTACCA
	2	GTGCGGCACTACAAGATCT
	3	ACGAGGCGGTGTCCTTCCT
siRNA ： LRRK2	1	GCATCATGGTTGAATGCTT
	2	GTACTCTCCTGGTCATCAA
	3	GCAACTGACTGAATTTGTT
